# Antagonistic control of myofiber size and muscle protein quality control by the ubiquitin ligase UBR4 during aging

**DOI:** 10.1038/s41467-021-21738-8

**Published:** 2021-03-03

**Authors:** Liam C. Hunt, Bronwen Schadeberg, Jared Stover, Benard Haugen, Vishwajeeth Pagala, Yong-Dong Wang, Jason Puglise, Elisabeth R. Barton, Junmin Peng, Fabio Demontis

**Affiliations:** 1 Department of Developmental Neurobiology, St. Jude Children’s Research Hospital, Memphis, TN USA; 2 Department of Structural Biology, Center for Proteomics and Metabolomics, St. Jude Children’s Research Hospital, Memphis, TN USA; 3 Department of Cell and Molecular Biology, St. Jude Children’s Research Hospital, Memphis, TN USA; 4 College of Health & Human Performance Applied Physiology & Kinesiology, University of Florida, Gainesville, FL USA

**Keywords:** Ubiquitin ligases, Ageing, Muscle

## Abstract

Sarcopenia is a degenerative condition that consists in age-induced atrophy and functional decline of skeletal muscle cells (myofibers). A common hypothesis is that inducing myofiber hypertrophy should also reinstate myofiber contractile function but such model has not been extensively tested. Here, we find that the levels of the ubiquitin ligase UBR4 increase in skeletal muscle with aging, and that UBR4 increases the proteolytic activity of the proteasome. Importantly, muscle-specific UBR4 loss rescues age-associated myofiber atrophy in mice. However, UBR4 loss reduces the muscle specific force and accelerates the decline in muscle protein quality that occurs with aging in mice. Similarly, hypertrophic signaling induced via muscle-specific loss of UBR4/poe and of ESCRT members (HGS/Hrs, STAM, USP8) that degrade ubiquitinated membrane proteins compromises muscle function and shortens lifespan in *Drosophila* by reducing protein quality control. Altogether, these findings indicate that these ubiquitin ligases antithetically regulate myofiber size and muscle protein quality control.

## Abstract (web summary)

Sarcopenia is the age-associated functional decline and atrophy of muscle fibers, and it has been proposed that it might be counteracted by inducing myofiber hypertrophy. Here, the authors show that expression levels of the ubiquitin ligase UBR4 are increased with ageing, and that whilst its genetic ablation rescues muscle atrophy, it is also associated with reduced protein quality and impaired force production in *Drosophila* and mouse models.

## Introduction

Skeletal muscle wasting is a debilitating condition associated with many human diseases but no therapy is currently available. Several studies have demonstrated that muscle wasting worsens disease outcome and decreases patient survival whereas preserving skeletal muscle mass and function is protective^1–3^. Previous studies have established that muscle wasting is typically determined by a decrease in myofiber size due to increased protein degradation versus protein synthesis^4^. Such myofiber atrophy primarily results from protein degradation via the autophagy–lysosome, and ubiquitin–proteasome systems^4,5^. In particular, transcriptional upregulation of several E3 ubiquitin ligases, including *Fbxo32* (atrogin-1/MAFbx), *Trim63* (MuRF1), *Fbxo30* (MUSA), and *Fbxo21* (SMART) has been reported to occur in atrophy and is considered responsible for poly-ubiquitin tagging and proteasomal degradation of target proteins during muscle wasting^4,5^.

In addition to disease-associated muscle wasting, age-related loss of muscle mass and function (sarcopenia) is an important medical issue. As for disease-associated muscle wasting, preventing age-related skeletal muscle dysfunction has important systemic consequences, and extends healthspan and longevity^6–8^. However, despite the important functions of ubiquitin ligases in disease-associated muscle wasting, the role that ubiquitin ligases have in sarcopenia is largely unexplored. It is frequently thought that sarcopenia is mediated by the same ubiquitin ligases and mechanisms responsible for disease-associated muscle wasting in the young. However recent research is inconsistent with this hypothesis^9^. Specifically, although the autophagy–lysosome, and ubiquitin–proteasome systems induce muscle atrophy in the young, their inhibition accelerates sarcopenia rather than protecting from it^6,9–13^. Similarly, mice that are null for the ubiquitin ligase FBXO32 (atrogin-1) experience a higher loss of muscle mass and strength during aging than do controls^14^, contrary to the expectation that they would be protected from sarcopenia. Moreover, although TRIM63 (MuRF1) knockout mice maintain muscle mass with age, they experience a higher decay rate of muscle strength than control mice^15^, suggesting that they are not protected from sarcopenia. Altogether, these findings suggest that ubiquitin ligases can exert contrasting effects on myofiber size and muscle strength depending on the age and/or persistence of their modulation. However, a mechanistic explanation for the deleterious effects deriving from the loss of these ubiquitin ligases in sarcopenia is missing. Moreover, because these studies have been limited to the analysis of few ubiquitin ligases, it remains unknown how sarcopenia is generally impacted by interventions based on ubiquitin ligases.

Through *Drosophila* genetic screening, we have previously probed the role that 323 evolutionary-conserved ubiquitin ligases play in myofiber size regulation^16^. We have found a set of ubiquitin ligases that are limiting for myofiber growth in *Drosophila* and mice, and that induce hypertrophy when their levels are decreased, including the ubiquitin ligase UBR4^16^. To probe the outcome of ubiquitin ligases in muscle aging in *Drosophila*, we examined the muscle-specific knockdown of 77 of such evolutionarily-conserved ubiquitin ligases that we previously uncovered as regulators of developmental myofiber growth^16^. We find that hypertrophic signaling induced in adult muscle via modulation of several ubiquitin ligases shortens lifespan in *Drosophila*, at least in part because of impaired protein quality control and reduced skeletal muscle function with aging. For example, we find that muscle-specific UBR4 RNAi reduces protein quality control, worsens age-related muscle functional decay, and shortens lifespan in *Drosophila*. Moreover, we find that muscle-specific UBR4 loss in old mice restores muscle mass at the expense of worsening muscle protein quality control and strength, although such decline in protein quality control induces an adaptive stress response based on the ubiquitin ligase CHIP, which is known to promote the degradation of misfolded proteins and to protect from sarcopenia^17,18^.

In summary, these findings indicate that, as exemplified by UBR4 loss, interventions that promote hypertrophy can have contrasting effects on the two components of sarcopenia, i.e., muscle mass and strength. Specifically, our studies indicate that the induction of myofiber hypertrophy occurs at the expense of compromising protein quality control and muscle function. Altogether, these studies highlight the importance of protein quality control for improving muscle strength in the aged. On this basis, we propose that therapies for sarcopenia should focus on preserving muscle protein quality control while attempting to reinstate muscle mass.

## Results

### Muscle-specific loss of UBR4 induces myofiber hypertrophy but this response is progressively reduced with aging

Previously, we have found that UBR4 loss induces myofiber hypertrophy in young mice^16^. Because aging is characterized by myofiber atrophy, we hypothesized that this may be rescued by UBR4 loss.

**Fig. 1:**
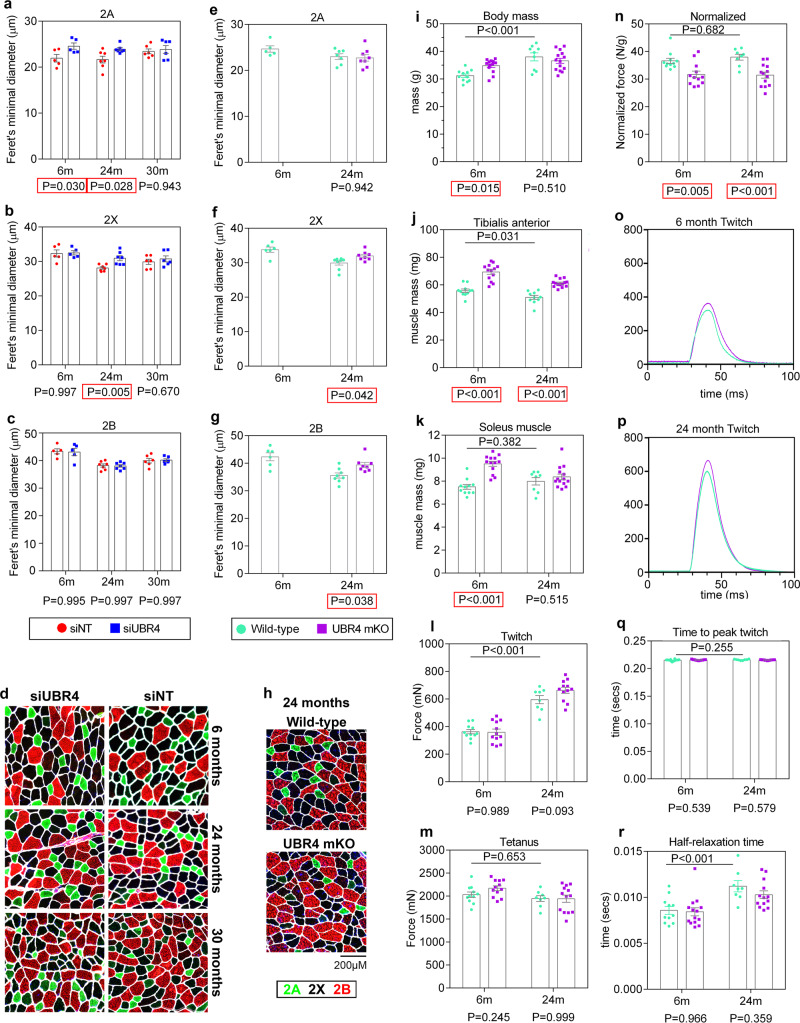
Muscle-specific loss of UBR4 rescues age-associated muscle and myofiber atrophy but decreases the normalized muscle force. **a**–**c** Quantitation of myofiber average sizes demonstrate age-dependent myofiber atrophy that is partially reversed in a myofiber type- and the age-dependent manner by UBR4 siRNAs; *n* = 5 (6-month-old mice), *n* = 7 (24-month-old mice), and *n* = 6 (30-month-old mice) biologically independent mice with matched muscles from contralateral legs are employed for comparison of NT siRNAs versus UBR4 siRNAs. **d** Representative images of tibialis anterior muscles immunostained for myosin heavy chain 2A (green) and 2B (red), with laminin (white) delineating the myofiber boundaries. **e**–**h** UBR4 muscle-specific knockout mice (UBR4 mKO; UBR4^fl/fl^ + ACTA-Cre) have increased myofiber size in old age compared to controls (UBR4^fl/fl^); *n* = 5 (6-month-old WT), *n* = 7 (24-month-old WT), and *n* = 7 (24-month-old UBR4 mKO) muscles from biologically independent mice. **i** UBR4 mKO show increased body mass and **j** tibialis anterior muscle mass at 6 months of age while maintaining greater tibialis anterior muscle mass at 24 months of age; *n* = 11 (6-month-old WT), *n* = 13 (6-month-old UBR4 mKO), *n* = 9 (24-month-old WT), and *n* = 14 (24-month-old UBR4 mKO) muscles from biologically independent mice. **k** Soleus muscle mass is increased at 6 months of age with UBR4 mKO but not at 24 months. **l** Twitch force of tibialis anterior muscle increases with age but is unaffected by UBR4 mKO. **m** Tetanic force is also unaffected by UBR4 mKO. **n** However, tetanic force normalized to muscle mass is reduced at all ages with UBR4 mKO. **o**–**p** Traces of twitch force produced over time in 6 and 24-month-old mice show no effect of UBR4 mKO but an increase with age. **q** No change in the time to peak twitch force but increased (**r**) half-relaxation time is found at 24 months compared to 6 months. In **l**–**r**, *n* = 11 (6-month-old WT), *n* = 12 (6-month-old UBR4 mKO), *n* = 8 (24-month-old WT), and *n* = 13 (24-month-old UBR4 mKO) muscle functional tests from biologically independent mice. Data are presented as mean ± SEM. All statistics were calculated by using two-way ANOVA with Tukey’s post hoc test with adjustment for multiple comparisons.

To test this hypothesis, a reduction in UBR4 levels was obtained via electroporation of siRNAs targeting UBR4 in the tibialis anterior muscle. Coincident with a decline in UBR4 mRNA levels (Supplementary Figure 1a), UBR4 siRNAs led to rapid induction of myofiber hypertrophy in young (6-month-old) mice (within 7 days from electroporation), compared to contralateral electroporation of control non-targeting (NT) siRNAs (Fig. 1a). On this basis, we next examined whether UBR4 siRNAs can reverse myofiber atrophy in old (24- and 30-month-old) mice. Strikingly, loss of UBR4 reinstated the size of type 2A and 2X myofibers of tibialis anterior muscles from 24-month-old mice to values close to those of young 6-month-old mice, whereas type 2B myofibers were not affected (Fig. 1a–d). However, there was no significant rescue of age-induced myofiber atrophy in 30-month-old mice (Fig. 1a–c), possibly due to the resistance to anabolic stimuli which is typically seen in old age in both mice and humans^12,19–22^.

Tamoxifen-induced muscle-specific UBR4 knockout mice (UBR4 mKO) were also used to further probe the consequences of UBR4 loss on sarcopenia. Coincident with a decline in UBR4 mRNA levels (Supplementary Fig. 1b) in the tibialis anterior muscles of UBR4 mKO mice at 6 and 24 months of age, there was an increase in the average size of type 2X and type 2B myofibers in old age (Fig. 1e–h). This indicates that chronic loss of UBR4 leads to hypertrophy of type 2X and 2B myofibers in old age, whereas acute loss of UBR4 via siRNA electroporation (Fig. 1a–d) or via short-term UBR4 knockout increases the size of type 2A and 2X myofibers (Fig. 1e–h). Therefore, these findings indicate that UBR4 loss differentially affects distinct myofiber types depending on the age of mice and on whether acute versus the chronic loss of UBR4 is induced.

As a consequence of the increase in myofiber size, UBR4 mKO mice were characterized by overall higher body mass at a young age (Fig. 1i). Moreover, analysis of multiple muscles confirms that UBR4 knockout affects skeletal muscles throughout the body (Supplementary Fig. 2), as expected based on the use of the *ACTA1-CRE* for inducing UBR4 ablation, as this tool targets all skeletal muscles in mice^23^.

Importantly, there was an increase in the size of the tibialis anterior muscles compared to wild-type controls in both young and old age (Fig. 1j), demonstrating that loss of UBR4 can increase muscle mass in old age, although the effect is blunted. There was also an increase in the mass of the soleus muscle in 6-month-old UBR4 mKO mice but such hypertrophy was missing in 24-month-old UBR4 mKO mice (Fig. 1k). Although body mass was not significantly different between genotypes at 24 months of age (Fig. 1i), the mass of tibialis anterior and soleus muscles normalized to total body mass indicated a dramatic drop in the proportion of lean mass with age in wild-type controls, whereas UBR4 mKO mice maintained a higher proportion of lean mass (Supplementary Fig. 1c, d).

Altogether, these findings indicate that muscle-specific loss of UBR4 induces myofiber hypertrophy but that this response is progressively reduced with aging, which presumably reflects intracellular anabolic resistance to hypertrophy.

### UBR4 loss reduces muscle-specific force during aging in mice

Because muscle force production normally correlates with muscle mass^4,24^, we next assessed whether muscle hypertrophy in UBR4 mKO mice leads to corresponding changes in muscle strength. To this purpose, in situ force production by the tibialis anterior muscles of UBR4 mKO mice was recorded at 6 and 24 months of age. The absolute twitch and tetanic forces were not significantly different at either age (Fig. 1l-m). However, the normalized force production was significantly reduced in UBR4 mKO mice at 6 and 24 months of age, indicating that the additional muscle mass does not cause a proportional increase in contractile function and that such functional deficit is not age-dependent (Fig. 1n).

Because twitch force increased with age, we examined more closely the twitch parameters by looking at representative traces (Fig. 1o, p). The time to peak twitch did not change (Fig. 1q) whereas the half-relaxation time increased with age (Fig. 1r) but was not modified by UBR4 loss. The increase in the half-relaxation time with age suggests an age-related decline in muscle function caused by changes in calcium handling^25^ that cannot be ameliorated by myofiber hypertrophy induced by UBR4 loss.

Altogether, muscle-specific loss of UBR4-induced hypertrophy; however this was blunted with increasing age and did not successfully improve muscle functional parameters altered with age. Moreover, normalized measurements of muscle force production indicate that UBR4 loss reduces muscle contractile functions. We, therefore, set out to examine the molecular changes occurring in UBR4-depleted muscle in order to gain a mechanistic understanding of the function of UBR4 during skeletal muscle aging.

### Transcriptome and proteome changes induced by UBR4 loss are blunted by aging

**Fig. 2:**
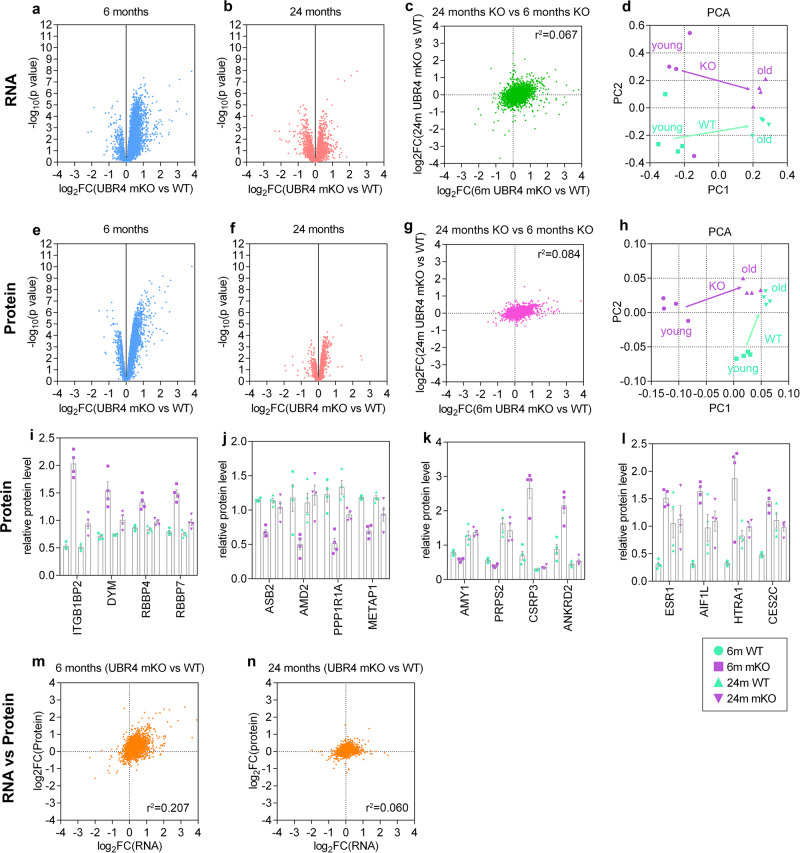
UBR4 loss causes RNA and protein changes in muscle that are diminished in magnitude with aging. **a** Volcano plots of RNA changes in UBR4 mKO compared to wild-type controls at 6 months and **b** 24 months of age. Significant changes are found in young age but the magnitude of such changes is reduced in old age, as also shown by the direct comparison of logarithmic fold changes between ages (**c**). **d** Principal component analysis (PCA) shows that old UBR4 mKO and wild-type samples cluster together while at 6 months they are further apart, indicating convergence towards a similar trajectory with aging. **e** Volcano plots of protein changes in UBR4 mKO compared to wild-type controls at 6 months and **f** 24 months show large changes in young age but limited changes in old age, as also shown by the direct comparison of log2FC values between ages (**g**). **h** A similar outcome is found by PCA analyses. **i**–**l** Changes in proteins that are unaffected by aging but upregulated with UBR4 mKO with diminishing magnitude in old age (**i**), unaffected by aging but downregulated with UBR4 mKO with diminishing magnitude in old age (**j**), upregulated by aging but downregulated by UBR4 mKO in young but not old age (**k**), and upregulated by aging and by UBR4 mKO in young age (**l**). **m**, **n** Comparison of RNA versus protein changes in young (**m**) and old age (**n**) shows decreased correlation between transcription and translation in old age in muscles from UBR4 mKO mice compared to controls. All data are from *n* = 3 (RNA-seq) and *n* = 4 (mass spectrometry) individual muscles from independent mice. Data are presented as mean ± SEM.

To gain mechanistic insight into the function of UBR4 during skeletal muscle aging, RNA sequencing and tandem mass tag (TMT)-based quantitative mass spectrometry were used to analyze the age- and UBR4-dependent changes that occurred in tibialis anterior muscles from 6 and 24-month-old UBR4 mKO mice and wild-type controls (Supplementary Data 1). Muscles from 6-month-old UBR4 mKO mice displayed many changes at the RNA and protein levels (Fig. 2a, e) that were consistent with those found in muscles with siRNA-mediated depletion of UBR4 (Supplementary Fig. 2a, b^16^). These included upregulation of proteins that we have previously shown to be necessary for myofiber hypertrophy induced by UBR4 loss^16^, such as ITGB1BP2, the histone chaperones RBBP4/7, and DYM (Fig. 2i). Interestingly, there were fewer transcripts and proteins that were significantly regulated by UBR4 loss at 24 versus 6 months of age (Fig. 2b, f). Consistently, the principal component analysis showed that UBR4 mKO muscles diverge less from the age-matched isogenic wild-type controls at 24 months of age, compared to what occurs at 6 months of age. Together, these findings indicate that the molecular changes induced by UBR4 loss at 6 months of age and responsible for the induction of hypertrophy are blunted at 24 months of age in parallel with a reduction in hypertrophy. These findings suggest that the effects of UBR4 depletion are reduced over time either through adaptive responses or age-related loss of sensitivity to UBR4 knockout.

Although there were several age-related changes in the transcriptome and proteome (Supplementary Fig. 2c, d) that were inversely affected by UBR4 mKO, overall there was a poor correlation between changes induced by UBR4 mKO and by age (Supplementary Fig. 2f–g). Similarly, there were subsets of proteins downregulated by UBR4 depletion at 6 months of age that were no longer downregulated at 24 months of age (Fig. 2j), including the atrophy mediator ASB2^26^. Thus, although the changes caused by UBR4 loss at a young age could potentially counteract age-induced changes, these UBR4-induced changes were mostly not maintained in old age (Fig. 2k), which may explain why the loss of UBR4 induces hypertrophy in young age but is less effective in old age. Finally, there were also proteins such as ESR1, AIF1L, HTRA1, and CES2C that were upregulated with aging as well as by UBR4 depletion in young age, indicating that UBR4 loss anticipates the occurrence of some age-induced changes (Fig. 2l). Taken together, this data indicate that UBR4 mKO is efficient at inducing molecular changes that coincide with hypertrophy at young age but that these are diminished in old age.

We recently found that the muscle transcriptome and proteome become progressively disconnected with aging in *Drosophila*^8^. By comparing the changes induced by UBR4 loss at the transcript level versus the protein level, we found an overall low correlation (*r*^2^ = 0.207) at 6 months of age, as expected based on the molecular function of UBR4, which is a ubiquitin ligase that modulates the degradation of target proteins, independently from their mRNA levels. However, this low protein-mRNA correlation is further reduced (*r*^2^ = 0.060) at 24 months of age (Fig. 2n and Supplementary Fig. 3), presumably due to age-induced defects in protein synthesis and degradation. These findings suggest that the combination of aging and UBR4 loss further disconnects the relationship between the transcriptome and the proteome more than UBR4 loss alone.

### UBR4 promotes protein degradation via the proteasome but not via the autophagy–lysosome system

Loss of UBR4 in mice increased muscle mass but reduced function (Fig. 1). Because muscle hypertrophy is typically characterized by a decrease in protein degradation^4,24^, we postulated that reduced muscle function associated with muscle hypertrophy could arise from the decreased capacity for protein turnover and for degrading misfolded proteins.

**Fig. 3:**
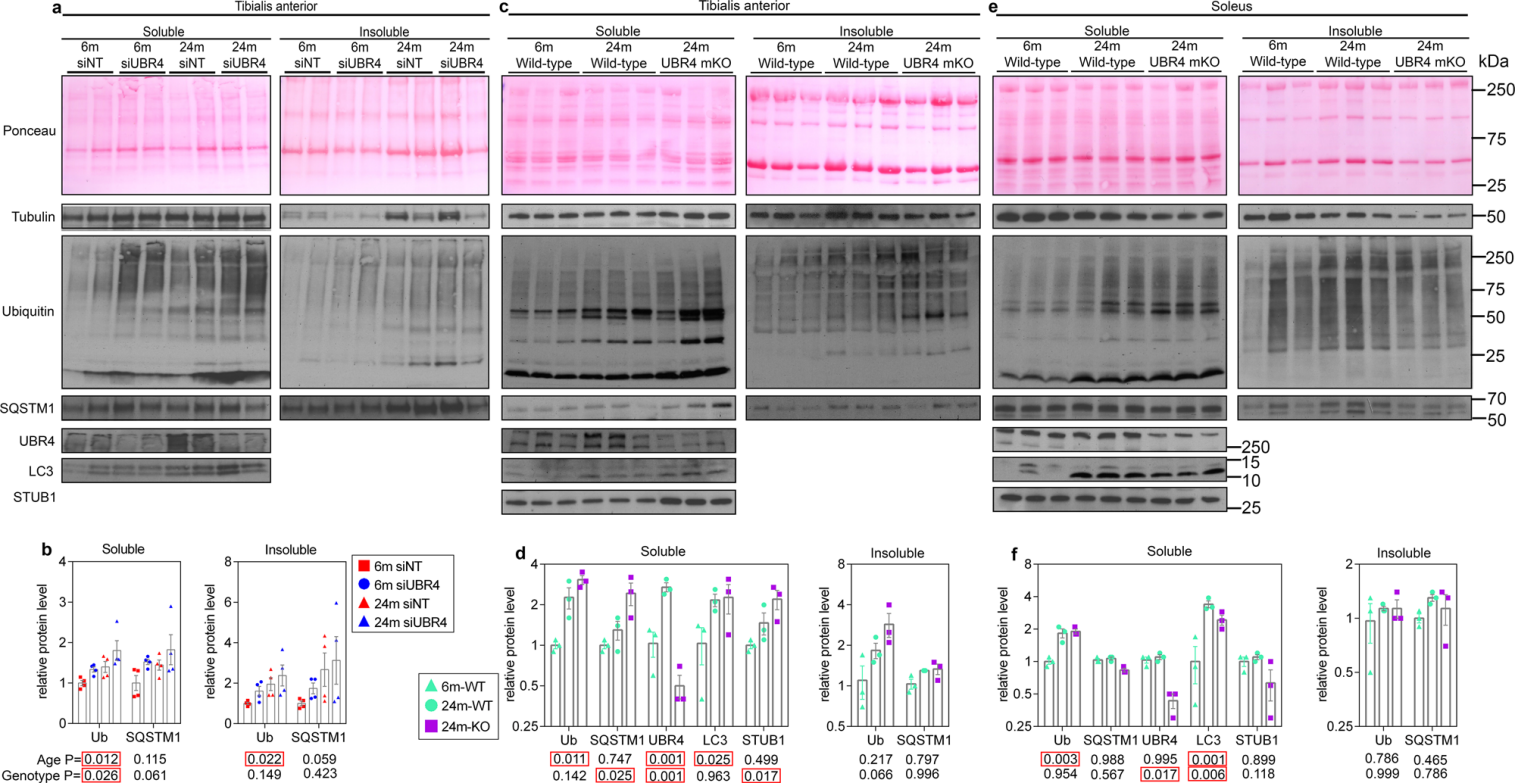
Defective protein quality control in aging skeletal muscles is exacerbated by UBR4 loss. Western blotting of detergent-soluble and insoluble proteins fractions from young and old tibialis anterior muscles with **a** UBR4 siRNA and from **c** UBR4 mKO mice, and **e** soleus muscles from UBR4 mKO mice, with quantitation, respectively, shown in **b**, **d**, and **f**. Ubiquitin and SQSTM1/p62 accumulate with age in both soluble and insoluble fractions of tibialis anterior muscles, and UBR4 loss leads to increased accumulation of these proteostasis markers. LC3 levels increase with age but are mostly unaffected by loss of UBR4. UBR4 loss increases STUB1/CHIP levels in the tibialis anterior but not in the soleus muscle. In the soleus, insoluble accumulation of ubiquitin and SQSTM1 is not evident, and only ubiquitin and LC3 accumulate in soluble fractions with age. Loss of UBR4 in the soleus muscle does not affect the accumulation of proteostasis markers, indicating that the soleus is better protected than the tibialis anterior muscle from proteostasis challenges that occur with aging. In **b**, *n* = 4 biologically independent mice with matched muscles from contralateral legs are employed for comparison of NT siRNAs versus UBR4 siRNAs. In **d**, **f**, *n* = 3 biologically independent muscles from separate mice. Data are presented as mean ± SEM. Statistics for **b**, **d**, and **f** were calculated by using two-way ANOVA with Tukey’s post hoc test with adjustment for multiple comparisons. Similar results were obtained from two independent sets of biological replicates.

To test this hypothesis, we, therefore, examined the levels of proteostasis markers in the muscles of UBR4 mKO mice. During aging, there was an increase in the levels of poly-ubiquitinated proteins in detergent-soluble and -insoluble fractions from the tibialis anterior muscle. Importantly, muscle-specific UBR4 loss increased the levels of poly-ubiquitinated proteins and SQSTM/p62 in the detergent-soluble fractions of tibialis anterior muscles. Autophagy also appeared altered with increased soluble LC3 protein in aged muscles, however, UBR4 loss had minimal effects on LC3 levels (Fig. 3a, c). Together, these findings indicate that proteostasis declines as muscles age and that UBR4 is required for maintaining muscle proteostasis. The soleus however demonstrated overall less changes with both age and UBR4 loss suggesting it is somewhat protected from proteostatic decline. Interestingly, UBR4 loss also led to the upregulation of *STUB1* (Fig. 3c), an E3 ubiquitin ligase (also known as CHIP), which promotes the degradation of misfolded proteins^27^, suggesting that it is part of a compensatory stress response that preserves muscle protein quality upon UBR4 loss. However, this occurred only in the tibialis anterior but not in the soleus muscle (Fig. 3e), indicating that CHIP is likely induced by loss of protein quality control and that this is not as pronounced in the soleus.

**Fig. 4:**
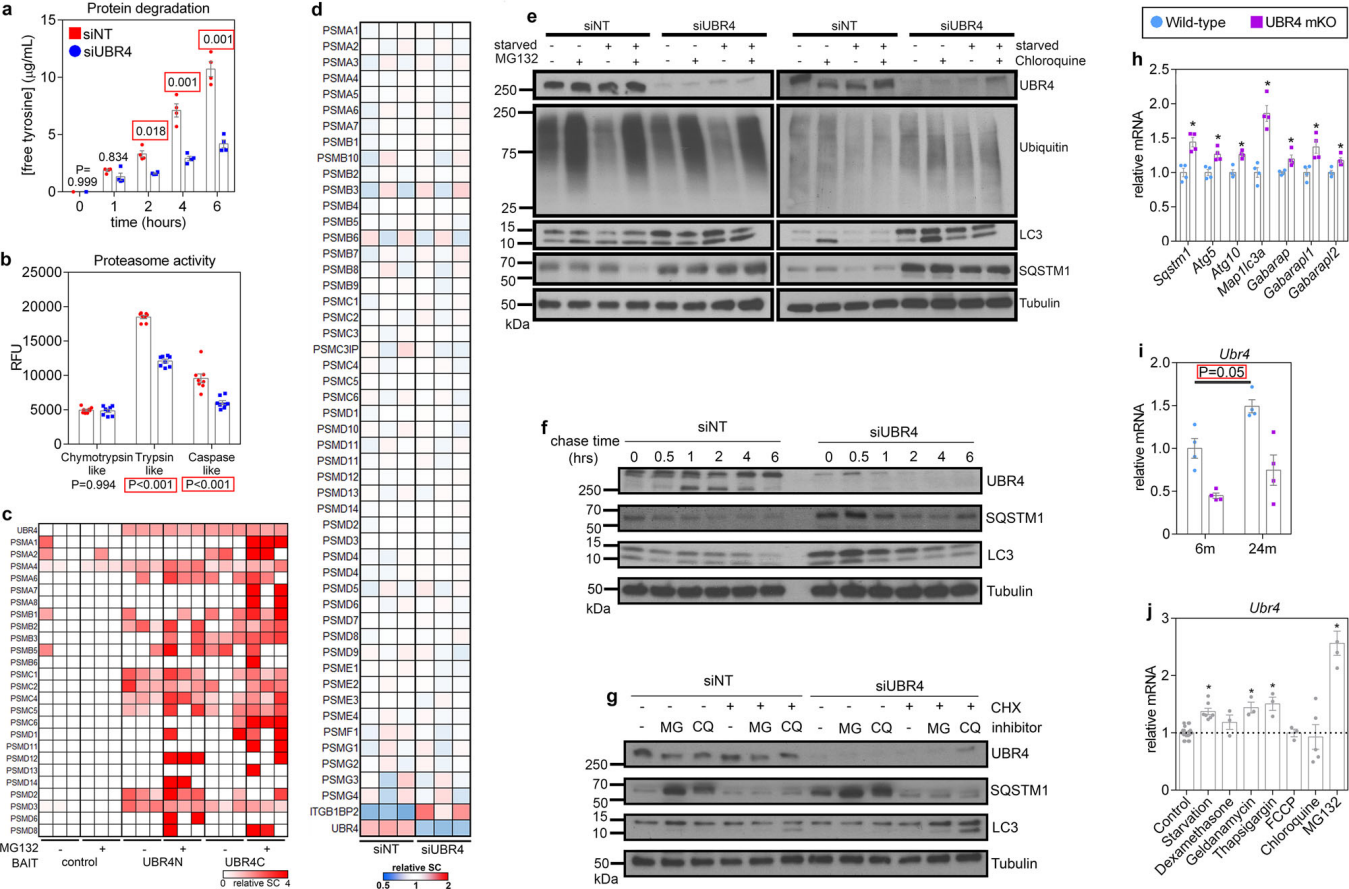
Loss of UBR4 compromises muscle protein quality control by inhibiting protein degradation by the proteasome. **a** Protein degradation rates measured by the release of free tyrosine over time in C2C12 cells are reduced by UBR4 knockdown; *n* = 4 biologically independent cell culture samples. **b** Trypsin and caspase-like proteolytic activities of the proteasome are reduced with UBR4 knockdown; *n* = 8 biologically independent cell culture samples. **c** Heatmap of spectral counts for proteasomal subunits that interact with N- and C-terminal UBR4 fragments (UBR4N and UBR4C) which were immunoprecipitated from cells treated with MG132 for 6 h. Data indicate a direct association between UBR4 and proteasome components, and this interaction further increases with proteasomal inhibition. **d** Heatmap which reports the protein levels of proteasomal subunits indicates that UBR4 siRNA does not change their levels (ITGB1BP2 and UBR4 are reported as positive controls). **e** Western blotting of cells that were short-term starved and treated with proteasome and lysosome inhibitors for 3 h in conjunction with UBR4 knockdown. Increased levels of the autophagic SQSTM1 and LC3 proteins are observed but no alterations in the autophagic flux are found when degradation pathways are stimulated with starvation or pharmacologically inhibited. **f** Western blots from pulse-chase experiments demonstrate increased starting levels of SQSTM1 and LC3 but similar degradation rates when cells are chased with the protein synthesis inhibitor cycloheximide under starvation conditions. **g** Increase in SQSTM1 protein levels caused by UBR4 loss or by inhibition of the proteasome (MG132 treatment for 8 h) is prevented by inhibiting protein synthesis with cycloheximide (CHX). **h** UBR4 loss increases the protein levels of SQSTM1, LC3, and other autophagic markers via their transcriptional upregulation. **i** UBR4 mRNA levels are upregulated in 24-month-old tibialis anterior muscles compared to 6-month-old. In **h**, **i** *n* = 4 independent muscles from separate mice. Statistical analysis for **a**, **b**, **i** was done by using two-way ANOVA with Tukey’s post hoc test with adjustment for multiple comparisons. **j** UBR4 expression increases in C2C12 cells exposed to proteostasis stressors for 24 h such as starvation and treatment with the Hsp90 inhibitor geldanamycin, the inducer of ER stress thapsigargin, and the proteasome inhibitor MG132; *n*(control) = 10, *n*(starvation) = 9, *n*(dexamethasone) = 3, *n*(geldanamycin) = 3, *n*(thapsigargin) = 3, *n*(FCCP) = 3, *n*(chloroquine) = 5, *n*(MG132) = 4 biologically independent cell culture samples for each group respectively; statistical analysis was done with one-way ANOVA with Dunnett’s post hoc test. Data are presented as mean ± SEM. For **a**, **b**, **e**–**g**, **j** similar results were obtained from two independent sets of biological replicates.

To gain further mechanistic insight into how UBR4 regulates protein quality control, we analyzed protein degradation rates in C2C12 cells depleted of UBR4. There was a significant reduction in the rate of protein degradation with UBR4 knockdown, as measured by free tyrosine release under catabolic conditions (i.e., starvation with PBS; Fig. 4a). Because protein degradation can be performed by both the lysosome and the proteasome, we next examined whether UBR4 regulates either of these proteolytic systems. Consistent with the role of UBR4 in modulating the function of the proteasome, UBR4 knockdown significantly reduced trypsin and caspase-like proteolytic activities of the proteasome (Fig. 4b). Because UBR4 is an E3 ubiquitin ligase, it is unclear how UBR4 would regulate the proteolytic activity of the proteasome. However, UBR4 has been previously found to associate with the proteasome independently of ubiquitin-binding, and this association further increases with proteasomal inhibition^28,29^. Consistently, our previous analysis of the UBR4 interactome^16^ indicates that UBR4 N- and C-terminal fragments co-immunoprecipitate with proteasomal subunits and that the frequency of such interactions increases upon proteasomal inhibition via MG132 (Fig. 4c) independently from changes in the expression of proteasomal subunits (Fig. 4d). Therefore, we suggest that UBR4 regulates proteasomal activity via direct interactions and not by regulating the expression of proteasome components.

Concerning the autophagy–lysosome system of protein degradation, UBR4 knockdown increases SQSTM1/p62 and LC3 levels, consistent with a previous report^30^. However, this increase in SQSTM1/p62 and LC3 levels was not altered in presence of short-term (3-h) proteasomal (MG132) or lysosomal (chloroquine) inhibition (Fig. 4e), indicating that may arise via processes such as protein synthesis that are independent of lysosomal and proteasomal degradation. To further test whether the autophagic flux of SQSTM1/p62 and LC3 proteins occurs at the same rate in UBR4-depleted versus control cells, we performed chase experiments in which protein synthesis was blocked for up to 6 h with cycloheximide and degradation stimulated with the starvation medium. These experiments indicated that SQSTM1/p62 and LC3 proteins are degraded at the same rate with a loss of UBR4, despite higher initial levels (Fig. 4f). Therefore, the initial increased levels of SQSTM1/p62 and LC3 proteins found in C2C12 cells depleted of UBR4 could be due to increased synthesis and not due to inhibition of their degradation. To test this hypothesis, we administered cycloheximide (CHX) for longer periods (24 h) to prevent protein synthesis and found that it prevented upregulation of SQSTM1/p62 and LC3 in response to the loss of UBR4, indicating that their levels are increased due to enhanced synthesis (Fig. 4g). Interestingly, loss of UBR4 in muscles increased the mRNA levels of several autophagy components, including SQSTM1/p62 and LC3 (Fig. 4h), indicating that increased expression of these genes may be responsible for the higher levels of SQSTM1/p62 and LC3 proteins found in response to UBR4 loss. Altogether these studies demonstrate that loss of UBR4 compromises muscle protein quality control by reducing proteasome-mediated proteolysis but not by impacting degradation via the autophagy–lysosome system.

### UBR4 is upregulated by aging and proteostatic stress

Because UBR4 regulates proteostasis, we examined whether UBR4 is modulated by proteostatic stressors. Interestingly, UBR4 mRNA (Fig. 4i) and protein levels (Fig. 3) were higher in the tibialis anterior muscle of 24- versus 6-month-old mice. However, this age-dependent increase in UBR4 levels was not found in the soleus (Fig. 3e) which appears resilient to proteostatic defects associated with aging. To further test the regulation of UBR4 by proteostatic challenges, proteostasis was perturbed in C2C12 cells via long-term starvation (8 h), geldanamycin (an inhibitor of Hsp90), thapsigargin (an inducer of endoplasmic reticulum stress), and MG132 (an inhibitor of proteasomal activity) treatment for 24 h (Fig. 4j). In all these cases, UBR4 mRNA levels were increased, especially with proteasomal inhibition. Altogether, these findings suggest that UBR4 levels may increase during aging due to age-associated challenges to proteostasis^9,12^, and that the resulting increased UBR4 activity may, in turn, promote myofiber atrophy with aging.

### UBR4 regulates protein quality control and age-related muscle functional decay in *Drosophila*

**Fig. 5:**
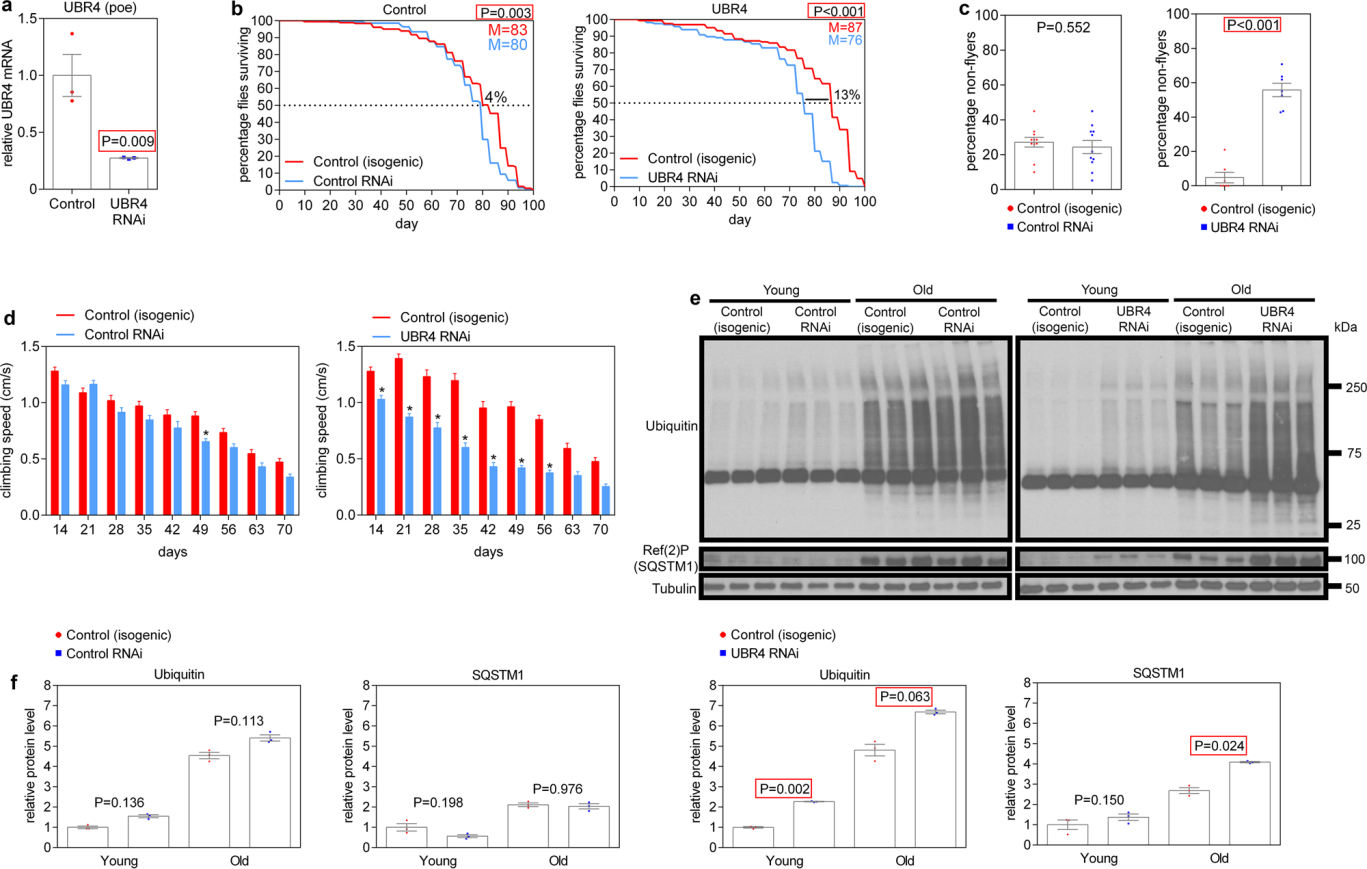
Muscle-specific loss of UBR4 compromises protein quality control and muscle function and shortens lifespan in *Drosophila*. **a** UBR4 knockdown measured by qRT-PCR from thoraces with UBR4 RNAi driven by the adult muscle-specific *MHC(F3.580)-GAL4*. **b** UBR4 loss in adult *Drosophila* muscle reduces the median lifespan by ~13% compared to isogenic controls whereas control RNAi has minimal effects (~4% reduction). In response to muscle-specific UBR4 loss, decreased muscle function was also observed, as indicated by the **c** flying ability and the **d** climbing speed. **e** Detergent-insoluble protein extracts showed increased levels of ubiquitin and Ref(2)P (*Drosophila* SQSTM1) in skeletal muscle with UBR4 knockdown. **f** Quantifications demonstrate compromised protein quality control in response to UBR4 loss; * indicates *P* < 0.05. In **a**, **f** *n* = 3 biological replicates, each consisting of 10 thoraces. In **b**, **d** *n* > 100 flies per group were used for the longitudinal survival and climbing speed analysis (specific *P* and *n* values are reported in the Source Data file). In **c** the percentage of flies not flying was determined for each individual tube of flies (each tube with ~20 flies), with *n*(control RNAi) = 10 and *n*(UBR4 RNAi) = 7. Data are presented as mean ± SEM. In **a**, **c** two-tailed Student’s *t*-test was used. In **b** the OASIS2 webtool and log-rank tests were used for the statistical analysis of lifespan. In **d**, **f** statistics were calculated by using two-way ANOVA with Bonferroni’s post hoc test with adjustment for multiple comparisons. Similar results were obtained from two independent sets of biological replicates.

To better examine the physiological consequences of UBR4 loss in skeletal muscle during aging, we next examined the function of UBR4 (*poe*) in *Drosophila*. For these studies, UBR4 mRNA knockdown was achieved (Fig. 5a) specifically in skeletal muscle via the UAS/Gal4 system and the adult muscle-specific *Mhc-Gal4.F3-580* driver^31^. Compared to its isogenic controls (*+/white*^*RNAi*^, i.e., transgene-alone controls), induction of a control white RNAi (*MhcF3* > *white*^*RNAi*^) had little effect on the median lifespan (∼4% decrease) and on muscle function, as estimated with negative geotaxis (climbing) and flight assays (Fig. 5b-d). However, UBR4 RNAi reduced the median lifespan by ∼13% (*MhcF3* > *Ubr4*^*RNAi*^), compared to its isogenic controls (*+/Ubr4*^*RNAi*^; Fig. 5b). Moreover, UBR4 RNAi also significantly reduced muscle function, as estimated with flight (Fig. 5c) and climbing (Fig. 5d) assays, compared to isogenic controls.

As we have found in mice (Fig. 3), also in *Drosophila* the decline in protein quality control is an important determinant of age-related skeletal muscle functional decay, which can be assessed by examining the detergent-insoluble protein aggregates^6^. Western blot analyses of detergent-insoluble fractions of fly thoraces (enriched in skeletal muscles) indicate that UBR4 RNAi increases the age-related accumulation of poly-ubiquitinated proteins and of the associated ubiquitin-binding protein p62/SQSTM1, *Drosophila* Ref(2)P (Fig. 5e, f).

**Fig. 6:**
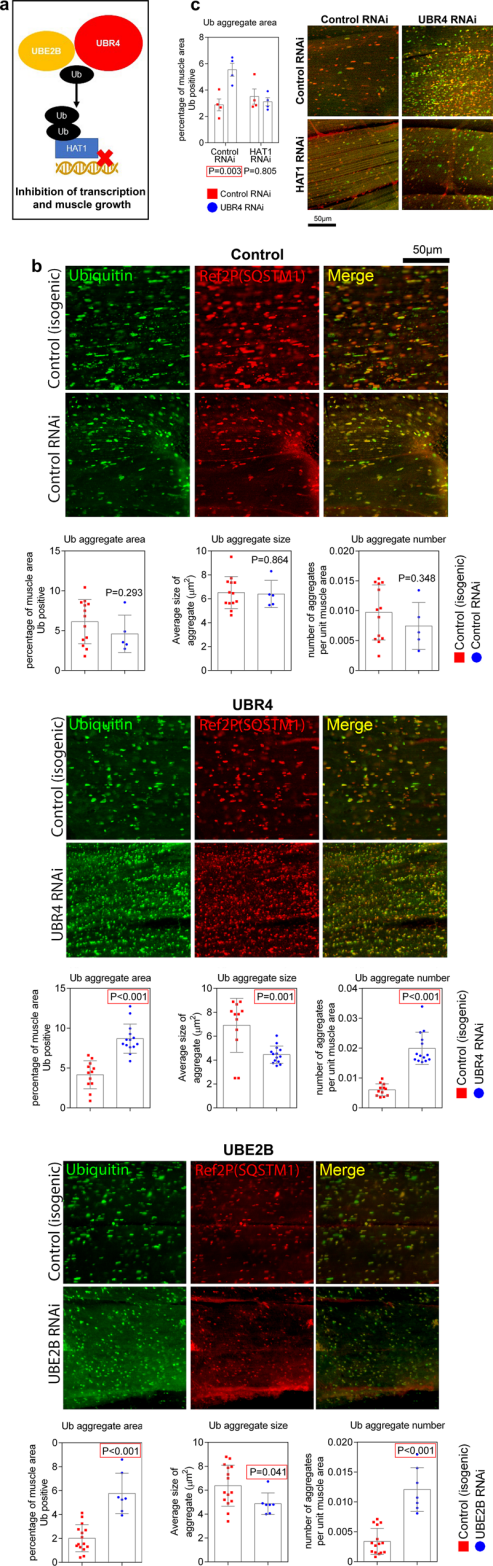
Muscle-specific loss of UBR4 compromises protein quality control and aggresome formation in *Drosophila* via its ubiquitination target HAT1. **a** Scheme that shows the ubiquitin ligase UBR4 working in concert with its associated ubiquitin-conjugating enzyme UBE2B. UBE2B/UBR4 target HAT1 for ubiquitin-dependent degradation to induce muscle hypertrophy, as identified in a previous study^16^. **b** Immunostaining of protein aggregates from indirect flight muscles at 35 days of age, with ubiquitin labeled green and SQSTM1 (ref2P) labeled red. The total area and number of ubiquitin and Ref(2)P-positive protein aggregates increases with loss of UBR4 and UBE2B during aging, whereas the average size of these aggregates decreases, indicating that their transport and clustering is perturbed by loss of UBR4 and UBE2B. **c** Increase in ubiquitin-protein aggregates induced by UBR4 loss is prevented by concomitant RNAi for HAT1, demonstrating that the same UBE2B/UBR4/HAT1 axis regulates myofiber hypertrophy^16^ also regulates protein quality control during aging. In **b** the *n* for isogenic control/RNAi comparisons is *n* = 13 and *n* = 5 for control RNAi, *n* = 12 and *n* = 14 for UBR4 RNAi, and *n* = 15 and *n* = 7 for UBE2B RNAi; each biological replicate consists of a hemithorax from independent flies. In **c** *n* = 4 biological replicates. Data are presented as mean ± SEM. All statistics were calculated by using two-way ANOVA with Tukey’s post hoc test with adjustment for multiple comparisons. Data are compiled from three individual sets of experimental fly groups collected and assessed separately but providing similar and comparable results.

Previously, UBR4 was identified to work together with the E2 ubiquitin-conjugating enzyme UBE2B to mediate ubiquitination and degradation of its substrate HAT1^16^, which is necessary for inducing myofiber hypertrophy (Fig. 6a). Immunostaining of skeletal muscle from 8-week-old flies indicates that UBR4 RNAi increases the total area and number of SQSTM1-positive aggregates of poly-ubiquitinated proteins, though reduces the individual aggregate size (Fig. 6b). Therefore, UBR4 is necessary for maintaining protein quality control in skeletal muscle during aging, which impacts muscle function and lifespan. Consistent with UBR4 acting together with UBE2B, loss of the *Drosophila* homolog of UBE2B (Ubc6) leads to a phenotype similar to that of UBR4 RNAi. Specifically, UBE2B RNAi leads to an increase in the total area and number of poly-ubiquitin protein aggregates and to a decrease in the size of individual aggregates (Fig. 5b). Furthermore, HAT1 is necessary for these phenotypes because HAT1 RNAi prevents the increase in poly-ubiquitin protein aggregates caused by UBR4 RNAi (Fig. 5c), compared to a control RNAi. Similar conclusions were reached also by examining the degradation of pathogenic huntingtin-polyQ in the *Drosophila* retina. Specifically, the UBE2B/UBR4/HAT1 axis regulates proteostasis also in this context as indicated by the increase in the aggregates of huntingtin-polyQ in response to UBR4 RNAi and the suppression of this phenotype by concomitant HAT1 RNAi (Supplementary Fig. 4). Conversely to what found with UBR4 RNAi, *UBR4* overexpression reduces huntingtin-polyQ aggregates in the retina, and lifespan is extended when UBR4 is overexpressed in adult skeletal muscle (Supplementary Fig. 5). Taken together, these findings indicate that the UBE2B/UBR4/HAT1 axis that regulates myofiber size^16^ is also an important determinant of protein quality control during aging.

### Ubiquitin ligases that limit developmental myofiber growth are necessary for muscle function and protein quality control during aging in *Drosophila*

In addition to UBR4, we previously identified a number of ubiquitin ligases and ubiquitin-related genes that regulate myofiber size^16^. On this basis, we examined their function during skeletal muscle aging in *Drosophila*.

**Fig. 7:**
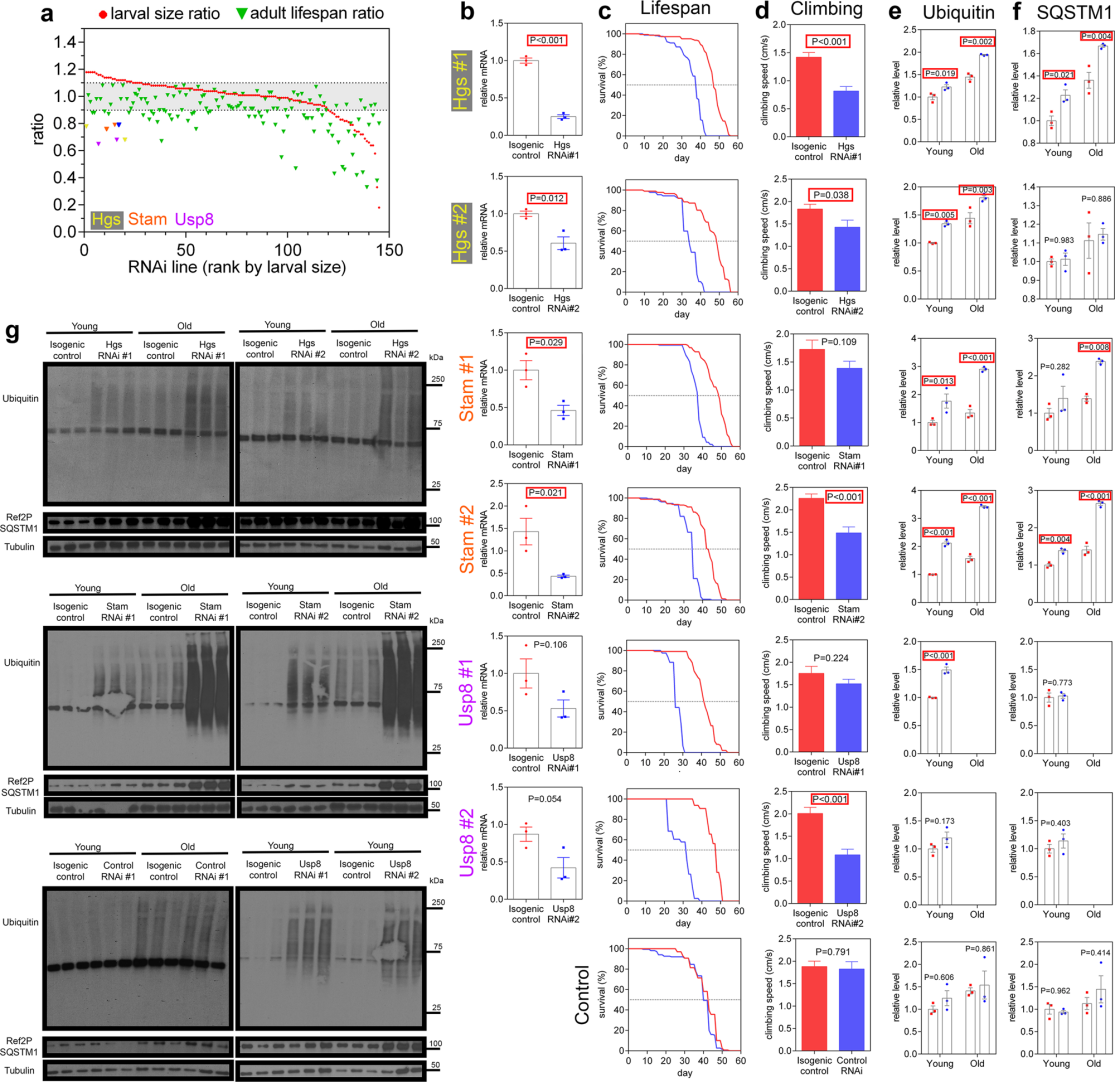
Ubiquitin ligases and related genes that negatively regulate myofiber size are positive regulators of muscle protein quality control, muscle function, and lifespan. **a** RNAi that regulates developmental myofiber growth with *Mef2-GAL4* (red dots) are examined for their capacity to regulate the median lifespan when expressed in adult muscle with *MHCF3-GAL4* (green triangles). In addition to UBR4, HGS, STAM, and USP8, many RNAi interventions that increased myofiber size during development reduced lifespan when modulated solely in adulthood (ratio of changes in the median lifespan compared to the isogenic control corresponding to each line). Median lifespan was derived from groups of >50 flies per RNAi line. **b** As expected, target genes are knocked down in the muscle of flies with muscle-specific RNAi driven by *MHCF3-GAL4*, compared to isogenic controls carrying the same transgenic RNAi but lacking *MHCF3-GAL4*. **c** Survival curves for HGS, STAM, and USP8 RNAi, each compared to its matched isogenic control. **d** Muscle function at 10 days of age is decreased as indicated by the reduced climbing speed compared to isogenic controls. For **c**, **d** information on *n* is provided in the Source Data file. **e**–**g** Muscle-specific knockdown of HGS, STAM, and USP8 increase the detergent-insoluble levels of ubiquitin and SQSTM1/Ref(2)P in thoracic skeletal muscles from young (10 days) and old flies (35 days) compared to isogenic controls whereas control RNAi had no effect compared to its isogenic control (note that flies with USP8 knockdown were short-lived and samples could not be obtained at old ages for these analyses). In **b**, **e**, **f** *n* = 3 biological replicates each consisting of 10 fly thoraces. Data are presented as mean ± SEM. In **b**, **d** two-tailed Student’s *t*-test was used. In **e**, **f** statistics were calculated by using two-way ANOVA with Tukey’s post hoc test with adjustment for multiple comparisons.

For these studies, we obtained an adult lifespan ratio by normalizing the median lifespan obtained from adult-onset muscle-specific RNAi (driven by *MhcF3-Gal4*) to the median lifespan of the corresponding isogenic transgene-alone control. Moreover, we also calculated a larval size ratio, which indicates the extent of myofiber growth induced by muscle-specific developmental RNAi expression (driven by *Mef2-Gal4*) normalized to the expression of a control RNAi (Supplementary Data 2). Compared to their respective isogenic transgene-alone controls, adult-onset muscle-specific RNAi for ubiquitin-related genes necessary for developmental myofiber growth shortened the median lifespan (Fig. 7a), suggesting that these ubiquitin ligases are essential for muscle cell viability during developmental growth as well as for adult muscle homeostasis.

We also examined the consequences of RNAi interventions that induce developmental myofiber hypertrophy. Interestingly, although hypertrophic signaling is usually considered a treatment for sarcopenia, we find that adult-onset muscle-specific induction of these RNAi interventions shortens the median lifespan, in comparison to isogenic controls (Fig. 7a). This is particularly evident for RNAi lines targeting the ubiquitin-binding proteins Stam and HGS/Hrs (here referred to as Hgs), the deubiquitinase Usp8, and the ubiquitin ligase UBR4/poe (Fig. 7b, c). Moreover, coincident with a decrease in their mRNA levels (Fig. 7b), the adult-onset induction of these RNAi does not protect from age-induced defects in skeletal muscle function but rather worsens muscle dysfunction with aging, as indicated by negative geotaxis (climbing) assays (Fig. 7d).

We next analyzed the detergent-insoluble fractions from thoraces, which consist primarily of skeletal muscles. Compared to a control RNAi and isogenic controls, RNAi for Hgs, Stam, Usp8, and UBR4 led to an increase in the detergent-insoluble levels of ubiquitin and SQSTM1/Ref(2)P (Fig. 7e–g), which is indicative of worsening of protein quality control with aging^6^. Thus, in addition to UBR4 RNAi, hypertrophic signaling induced by RNAi for other ubiquitin ligases also fails to prevent age-related muscle functional decay but rather worsens protein quality control during aging. Altogether, these findings suggest that worsening of protein quality control is a pervasive deleterious effect generally induced by hypertrophic signaling, as also seen with modulation of insulin/IGF signaling (Supplementary Fig. 6a). However, as estimated from the levels of phosphorylated Akt (Supplementary Fig. 6a), these ubiquitin ligases do not appear to alter insulin/IGF signaling, suggesting that they induce myofiber hypertrophy and impact protein quality control via Akt-independent mechanisms.

### Differential roles of ubiquitin ligases in the degradation of aggregation-prone proteins during muscle aging in *Drosophila*

**Fig. 8:**
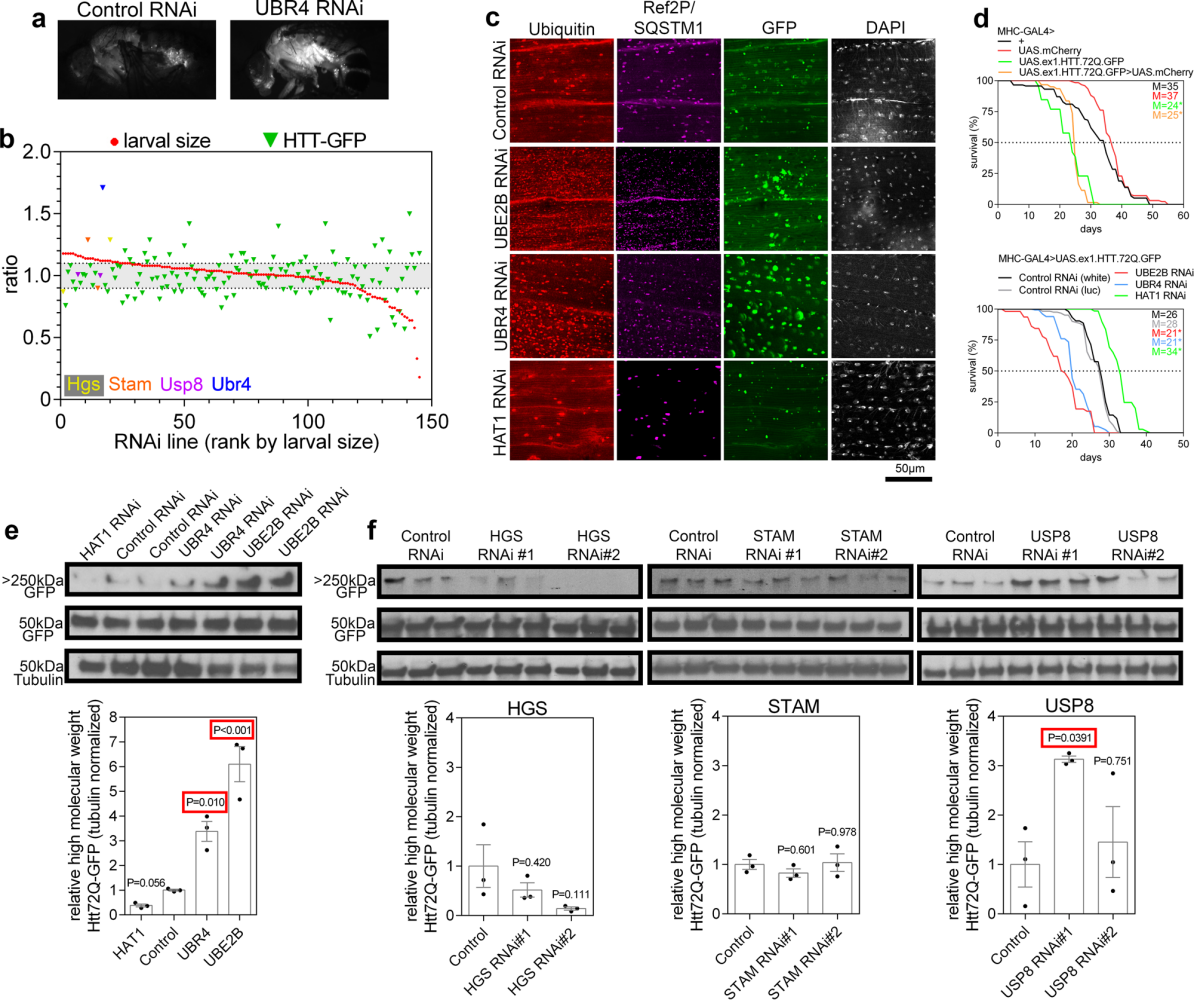
The UBE2B/UBR4/HAT1 axis regulates the degradation of pathogenic huntingtin-polyQ whereas other ubiquitin-related proteins do not. **a** Example of GFP fluorescence from GFP-tagged huntingtin-72Q (ex1.Htt.72Q.GFP) expressed in skeletal muscle. Note that the overall GFP fluorescence (indicative of Htt.72Q.GFP) increases with UBR4 knockdown. **b** Unlike UBR4, other ubiquitin ligases and related proteins that regulate protein quality control during aging do not consistently impact Htt-72Q-GFP aggregation, as estimated based on the GFP fluorescence intensity (the normalized fluorescence average from 10 individual flies is shown for each RNAi). **c** Immunofluorescence of Htt-72Q-GFP in thoracic indirect flight muscle. RNAi for UBR4 and UBE2B increases ubiquitin- and SQSTM1-positive protein aggregates, as well as Htt-72Q-GFP aggregates whereas HAT1 RNAi, reduces them. Ubiquitin is labeled red, SQSTM1 (ref2P) is purple, GFP is green, and DAPI is white. **d** Htt-72Q-GFP expression in adult skeletal muscle shortens the median lifespan (M), which is exacerbated by RNAi for UBE2B and UBR4, and inhibited by HAT1 RNAi; for each group, *n* = 116/107/126/108 flies for the top panel and *n* = 103/106/122/135/108 for the bottom panel, respectively. **e** Analyses by western blots detect an increase in high-molecular weight Htt-72Q-GFP aggregates with RNAi for UBE2B and UBR4 RNAi, whereas HAT1 RNAi reduces aggregation. **f** Western blotting for high-molecular weight Htt-72Q-GFP aggregates shows no increase with knockdown of HGS and STAM and inconsistent effects with USP8 RNAi. In **e**, **f** *n* = 3 biological replicates each consisting of 10 individual fly thoraces per biological replicate. Data are presented as mean ± SEM. In **d** the OASIS2 webtool and log-rank tests were used to calculate the difference between mean lifespans, with *P < 0.005 compared to control. In **e**, **f** statistics were calculated by using two-way ANOVA with Tukey’s post hoc test with adjustment for multiple comparisons.

Hypertrophic signaling leads to the age-related accumulation of markers of defective proteostasis, such as ubiquitin, SQSTM1/p62 (*Drosophila* Ref(2)P), and LC3 (*Drosophila* Atg8). To understand whether the inverse relationship between hypertrophic signaling and protein quality control applies generally to proteostatic challenges beyond aging, we tested a model of pathologic protein aggregation due to huntingtin-polyQ (Htt-72Q-GFP). For these studies, Htt-72Q-GFP was expressed in the skeletal muscle of adult flies and protein aggregates visualized by fluorescence microscopy. Loss of UBR4 increased Htt-72Q-GFP aggregates in thoracic indirect flight muscles, as estimated based on the immunofluorescence of whole flies (Fig. 8a). Regulators of growth were again screened for Htt-72Q-GFP aggregate accumulation (Fig. 8b). Similar to its effects on the poly-ubiquitin protein aggregates in aged muscle, the UBE2B/UBR4/HAT1 axis regulated aggregation of pathogenic Htt-72Q-GFP. Specifically, UBE2B RNAi and UBR4 RNAi increased Htt-72Q-GFP aggregation whereas HAT1 RNAi decreased it (Fig. 8c). Western blot analyses of detergent-insoluble fractions further revealed that high-molecular-weight Htt-72Q-GFP aggregates were also increased in muscle with UBR4 RNAi and UBE2B RNAi and, conversely, they were decreased with HAT1 RNAi (Fig. 8e).

Consistent with the role of muscle protein quality control in determining lifespan, expression of aggregation-prone *Htt-72Q-GFP* in muscle shortens lifespan^6^, and this was further reduced with UBR4 RNAi and UBE2B RNAi, which increased Htt-72Q-GFP aggregates (Fig. 8d). Conversely, lifespan was extended with HAT1 RNAi, which decreased aggregation (Fig. 8d). The same effects on protein aggregation can be seen when driving expression of *Htt-72Q-GFP* in the retina (Supplementary Fig. 4), indicating that UBE2B/UBR4/HAT1 regulates the aggregation of pathogenic proteins in other tissues besides muscle.

Interestingly, not all hypertrophic regulators influenced Htt-72Q-GFP aggregation in the same manner as UBE2B/UBR4/HAT1. In addition to no apparent increase in Htt-72Q-GFP fluorescence levels in the screen (Fig. 8b), western blots of detergent-insoluble fractions revealed that RNAi for HGS and STAM did not increase Htt-72Q-GFP aggregation (Fig. 8f). Because the levels of fluorescent Htt-72Q-GFP aggregates in muscles correspond to the overall GFP fluorescence of these flies (Fig. 8a, b), we used this readout to test whether RNAi interventions increase or decrease developmental myofiber growth predictably modulate Htt-72Q-GFP aggregation when induced in adult muscles. As expected, inhibition of the growth-promoting Insulin receptor signaling pathway (via RNAi for PDK1 and AKT) reduced Htt-72Q-GFP aggregation whereas its activation (via RNAi for PTEN) increased it (Supplementary Fig. 6b). However, we find that not all RNAi targeting ubiquitin ligases that increase developmental myofiber growth consistently modulate Htt-72Q-GFP aggregation (Fig. 8b). Overall, these findings indicate that hypertrophic signaling induced by distinct ubiquitin ligases and signaling pathways may differentially affect proteostasis, depending on the disease context and aggregation-prone protein.

### UBR4, HGS, STAM, and USP8 impact protein quality control via modulation of common and divergent protein targets

**Fig. 9:**
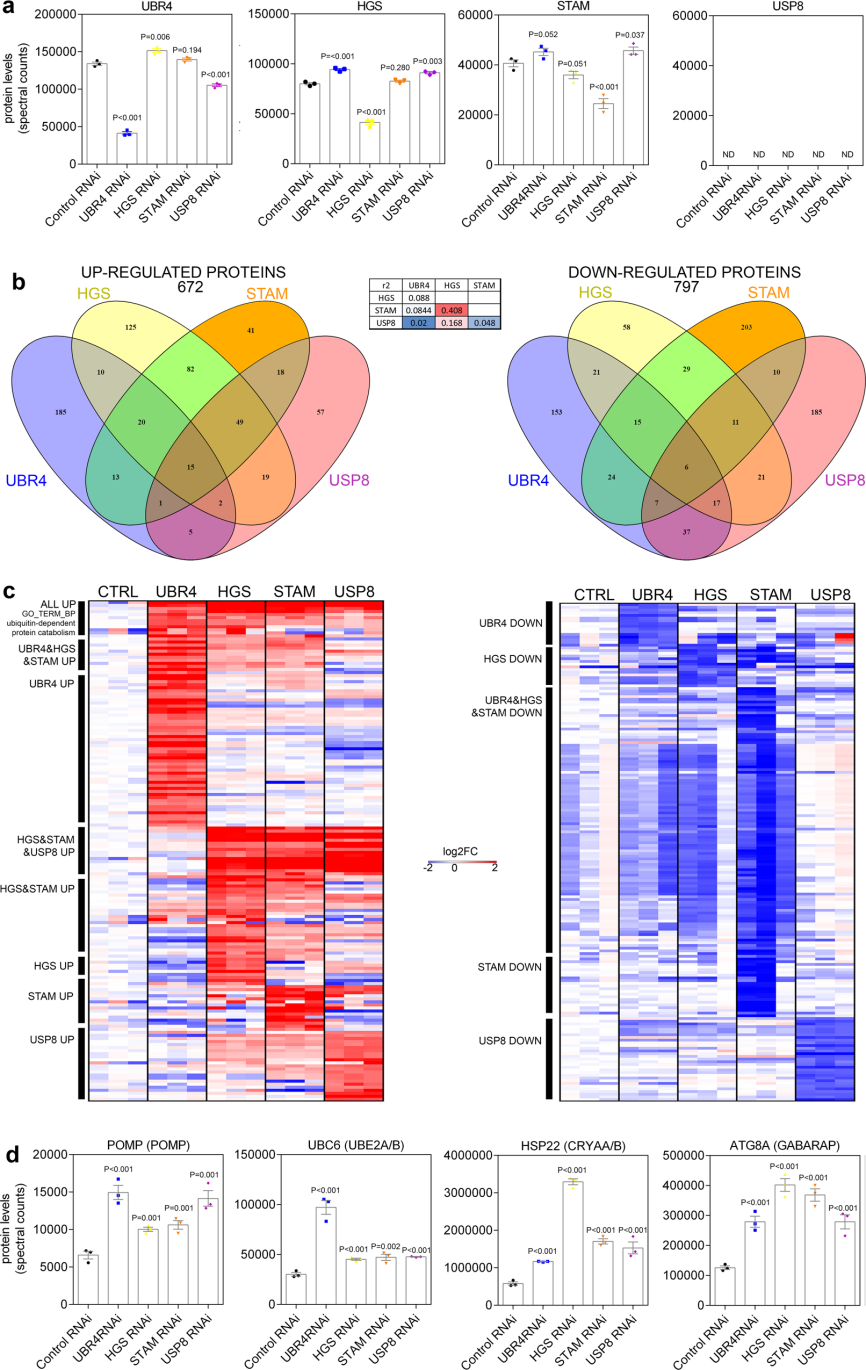
Loss of UBR4, HGS, STAM, and USP8 lead to a common proteomic signature of proteostatic stress through regulation of similar and divergent protein targets. **a** TMT-MS proteomics was performed to determine protein changes induced by RNAi targeting UBR4, HGS, STAM, and USP8 in skeletal muscles of adult *Drosophila*, compared to control RNAi; significant protein knockdown was confirmed for UBR4, HGS, and STAM; USP8 was not detected (ND) by this method. **b** Overall representation of significantly (*P* < 0.05) upregulated (log_2_FC > 0.5) and downregulated proteins (log_2_FC < −0.5) in response to RNAi for UBR4, HGS, STAM, and UPS8, compared to control RNAi. The inset table reports *r*2 values for RNAi-RNAi comparisons. Unique sets of proteins are modulated by each RNAi whereas there is only a limited overlap of protein targets. Specifically, there are 15 proteins consistently upregulated by RNAi for UBR4, HGS, STAM, and UPS8, and these include promoters of proteostasis such as POMP, UBC6, HSP22, and ATG8A (highlighted in **c** and **d**). However, proteins upregulated by RNAi for HGS and STAM display a higher degree of overlap, consistent with the shared function of HGS and STAM in the ESCRT complex and the endocytic degradation of ubiquitinated membrane proteins. **c** Heatmaps of 200 proteins that are significantly regulated (*P* < 0.05) by each RNAi with the highest magnitude of changes. The proteins commonly regulated by all RNAi are shown on top, with examples presented in panel d These heatmaps show that RNAi for UBR4, HGS, STAM, and UPS8 leads to upregulation of shared and distinct target proteins (left) whereas downregulated proteins (right) are mostly distinct. **d** Examples of proteostasis markers that are commonly upregulated by loss of UBR4, HGS, STAM, and USP8. These include the proteasome assembly factor POMP, the ubiquitin E2 conjugating enzyme UBC6 (homologous to mouse UBE2A/B), the chaperone HSP22 (homologous to mouse CRYAA/B), and the autophagy protein ATG8A (homologous to GABARAP/LC3). These promoters of proteostasis are presumably part of an adaptive response to limit the loss of protein quality control induced by loss of UBR4, HGS, STAM, and UPS8. *N* = 3 biological replicates consisting of pools of 50 individual fly thoraces per biological replicate. Data are presented as mean ± SEM. Statistical analysis was performed by one-way ANOVA with Benjamini–Hochberg false discovery testing for multiple comparisons.

To identify the mechanisms involved in the reduction of protein quality control due to loss of ubiquitin ligases, tandem mass tag (TMT) mass spectrometry was performed to identify proteins regulated in response to RNAi for UBR4, HGS, STAM, and USP8 in adult skeletal muscles (full dataset available in Supplementary Data 5). As expected based on the qRT-PCR analysis of mRNA levels (Fig. 7b), RNAi led to a reduction in the protein levels of UBR4, HGS, and STAM (Fig. 9a), whereas USP8 was not detected by TMT mass spectrometry. Altogether, RNAi for UBR4, HGS, STAM, and USP8 led to the upregulation of 672 proteins and to the downregulation of 797 proteins, compared to a control RNAi (white RNAi). A large proportion of the RNAi-modulated proteins, particularly downregulated proteins, were modulated in an RNAi-specific manner (Fig. 9b, c). However, there was a more substantial overlap between the changes in protein abundance induced by RNAi for HGS and STAM, which are components of the endosomal sorting complex required for transport (ESCRT) pathway, which is required for the ubiquitin-dependent degradation of membrane proteins^32^. Interestingly, UBR4 and USP8 have also been implicated in endocytic pathways^33–35^. USP8 was suggested to de-ubiquitinate proteins involved ESCRT (both cargo and integral components) and showed some overlap in regulated proteins with HGS and STAM, while UBR4 was proposed to influence endosomal biogenesis and also shares some regulated proteins with HGS and STAM. Altogether this may suggest some commonality between the role of these proteins and overall effects on proteostasis despite differing protein functions and targets. Overall, there were 15 proteins that were similarly upregulated by RNAi for UBR4, STAM, HGS, and USP8, compared to a control RNAi. These upregulated proteins included POMP (which is involved in proteasomal assembly), UBC6 (the E2 ubiquitin-conjugating enzyme that associates with UBR4), HSP22 (a chaperone involved in protein folding), and ATG8A, the autophagy protein homologous to LC3 and involved in the formation of autophagosomes (Fig. 9d). Taken together, this proteomic analysis indicates that UBR4, HGS, STAM, and USP8 impact protein quality control via unique sets of target proteins but that a common set of promoters of proteostasis is upregulated upon their loss, presumably as part of an adaptive mechanism to mitigate for the loss of protein quality control.

Finally, to provide a comprehensive analysis of the target proteins modulated by UBR4, HGS, STAM, and UPS8, we examined the gene ontology (GO) terms that were enriched for the set of proteins regulated by each RNAi (Supplementary Data 6). Some commonly enriched GO terms for upregulated protein were ubiquitin-dependent degradation (UBR4, HGS, and STAM), which included proteins involved in ubiquitination as well as proteasomal components, glutathione metabolism (UBR4, HGS, STAM, and USP8), and determination of adult lifespan (UBR4, HGS, STAM, and USP8). Thus, the loss of each of these genes produces similar molecular changes indicative of compromised muscle protein quality control and accelerated aging, consistent with Fig. 7. However, the enriched GO terms related to cellular components differed. Specifically, UBR4 RNAi primarily affected mitochondrial, cytosolic, and nuclear proteins; HGS and STAM affected plasma membrane proteins, consistent with their role in endocytosis; and USP8 affected plasma membrane, extracellular and lysosomal proteins. This analysis indicates that the direct targets of UBR4, HGS, STAM, and USP8 differ despite a similar outcome of their knockdown on protein quality control.

We also tested the evolutionary conservation of protein targets modulated by UBR4 loss in *Drosophila* versus mouse skeletal muscle. Remarkably, many homologous target proteins were similarly modulated in *Drosophila* and mice, including Ubc6/UBE2A, HAT1, CHORD/ITGB1BP2, Ufd4/HECTD1, and several others (highlighted in Supplementary Fig. 7 and full dataset comparison in Supplementary Data 7). This demonstrates that the function and mechanisms of action of UBR4 are overall conserved across species. However, certain proteins were notably upregulated in mice but not in *Drosophila* upon loss of UBR4. These included STUB1/CHIP, an E3 ubiquitin ligase that degrades misfolded proteins and which is upregulated in mice upon UBR4 loss, presumably as part of a compensatory response to the loss of protein quality control. The lack of STUB1/CHIP upregulation in *Drosophila* suggests that flies are less capable of adapting to challenges to muscle proteostasis. Taken together, the proteomic analysis of changes induced by RNAi for UBR4, STAM, HGS, and USP8 provides insight into common and RNAi-specific responses that affect muscle protein quality control and homeostasis.

## Discussion

In this study, we have used an extensive series of genetic interventions to test the long-standing hypothesis that promoting muscle growth should cure sarcopenia, i.e., the age-related loss of muscle mass and strength. Contrary to this common model, we find that hypertrophic signaling (induced via modulation of several ubiquitin ligases, including UBR4), while effectively increasing muscle mass, impairs muscle protein quality control and that this, in turn, reduces muscle function and shortens lifespan. Our finding that positive regulators of developmental myofiber growth negatively impact muscle protein quality control during aging is in agreement with the antagonistic pleiotropy theory of aging^36,37^, which posits that traits that are beneficial to the organism’s fitness in young age become detrimental in old age. Therefore, although muscle hypertrophy is considered a desirable trait at young age, muscle hypertrophic signaling does not appear to prevent muscle functional decline with aging despite its capacity to antagonize the age-related loss of muscle mass. Specifically, hypertrophic signaling may actually worsen age-related muscle dysfunction by compromising protein quality control.

Our findings are in line with mounting evidence that interventions that stimulate muscle growth and prevent atrophy in the young are detrimental for sarcopenia^9,12,24^. For example, increased activity of AKT, a transducer of the insulin/mTOR pathway, increases muscle mass but conversely reduces muscle function in aged mice^14^ whereas treatment with rapamycin, which inhibits mTOR, protects from sarcopenia in rats^38^. Moreover, inhibition of myostatin (a TGF-β ligand that negatively regulates muscle growth) increases muscle mass but reduces muscle force production and leads to the appearance of age-related ultrastructural defects, such as tubular aggregates^39,40^. However, not all hypertrophic stimuli reduce muscle function in the aged. Specifically, IGF-1 has been shown to effectively promote hypertrophy of aged muscle without decreasing specific muscle function^41,42^. This effect may be due to maintenance of regenerative capacity and/or compensatory activation of AMPK and autophagy^43^, which other hypertrophic stimuli may not be able to induce.

Another set of studies suggest that interventions that limit muscle growth may actually preserve muscle function with aging. For example, overexpression of *FoxO* transcription factors induces atrophy in mouse myofibers at young age^44^ as observed in *Drosophila* during development^24,45^. However, *FoxO* overexpression in the skeletal muscle of adult flies promotes protein quality control, reduces age-related muscle functional decline, and increases lifespan^6^. Similarly, overexpression of *myoglianin*, the *Drosophila* homolog of myostatin and GDF11, also inhibits muscle functional decline with age and increases lifespan^46^. Importantly, these interventions negatively regulate myofiber size during development in *Drosophila* but do not modulate myofiber size and muscle mass when targeted only to adult muscles (Supplementary Fig. 8). Therefore, this suggests that adult skeletal muscles are not particularly plastic with respect to changes in size in *Drosophila*^47^, and that growth signaling in the muscles of adult *Drosophila* predominantly influences protein quality control, muscle strength, and lifespan.

By targeting RNAi to the skeletal muscle of adult *Drosophila*, here we have examined the outcome of hypertrophic signaling induced by an extensive series of genetic interventions that modulate ubiquitin ligases and ubiquitin-related genes. Our extensive analysis supports the hypothesis that hypertrophic signaling is generally detrimental to the maintenance of skeletal muscle strength during aging. Specifically, we have screened 144 RNAi for ubiquitin ligases that regulate muscle growth and demonstrate that hypertrophic signaling is consistently and inversely correlated with muscle protein quality control. Therefore, hypertrophic signaling generally worsens muscle protein quality control and function during aging. However, such effects do not seem to stem from a convergence in modulating AKT signaling (based on the analysis of AKT phosphorylation), suggesting that different growth-promoting targets and signaling pathways are modulated by distinct ubiquitin ligases, as demonstrated by our proteomic analyses. Specifically, RNAi for UBR4, HGS, STAM, and USP8 (which induces myofiber hypertrophy during development) reduces protein quality control, muscle function, and lifespan via impacting some overlapping and some distinct protein targets. Interestingly though all of these proteins have distinct functions (with the exception of HGS and STAM that form the ESCRT-0 component^32^), they have all been implicated in endocytosis^33–35^. Although our proteomic analysis agrees that HGS and STAM have a large overlap in function, UBR4 and USP8 individually also appear to share some overlap with HGS and STAM, and regulation of endocytosis may constitute a shared mechanism for modulating growth and protein quality control. However, we find that HGS, STAM, USP8, and UBR4 also modulate distinct protein targets. For example, loss of UBR4 induces myofiber hypertrophy through reduced degradation and accumulation of HAT1, which we find to be important also for modulating proteostasis. Interestingly, HAT1 is upregulated also by USP8 loss but it is not regulated by RNAi for HGS, STAM. Therefore, ubiquitin ligases that regulate myofiber hypertrophy impact hypertrophic signaling and protein quality control largely via specific sets of target proteins.

In addition to the analyses in *Drosophila*, we have also examined the function of UBR4 in mice and have found that UBR4 has an evolutionary-conserved role in regulating muscle protein quality control during aging. Previously, it has been demonstrated that UBR4 is an E3 ubiquitin ligase that promotes the degradation of specific target proteins by the proteasome^16,48–50^, with which UBR4 physically associates^28,29^. Moreover, UBR4 loss has also been shown to increase the levels of autophagy components^30^, the aggregation of polyQ huntingtin protein^51^, and modulate the endocytic trafficking of cell surface proteins^33^. However, this previous knowledge does not explain how UBR4 modulates protein quality control.

Our study now provides key mechanistic insight into the role of UBR4 in protein quality control. Specifically, we have found that UBR4 directly binds to proteasomal components and regulates the proteolytic activity of the proteasome. Consistent with a previous report^30^, we find that UBR4 loss increases the levels of p62 and LC3 also in skeletal muscle but we determine that such changes are caused by increased transcription and translation of SQSTM1/p62 and LC3 rather than by inhibition of the autophagic flux. Therefore, we conclude that UBR4 promotes proteolysis via the proteasome but not via the autophagy–lysosome system in skeletal muscle. Consistently, we have found that UBR4 loss in *Drosophila* accelerates the age-related decline in protein quality control, as exemplified by the higher number and total area of poly-ubiquitin protein aggregates. UBR4 loss also leads to a decrease in the size of such protein aggregates, which is possibly related to the endocytic trafficking defects previously observed in other systems upon UBR4 loss^33^. Moreover, we have identified ubiquitination target proteins that are modulated by UBR4, including HAT1, that contribute to the regulation of protein quality control in the context of normal aging and of pathogenic Htt-polyQ aggregation.

In addition to reducing proteasome activity and protein quality control, we found that UBR4 loss decreases muscle function in flies and mice. Specifically, UBR4 loss reduced the normalized muscle force in mice, which could affect the execution of complex movements by those muscles, particularly in old age. Such muscle functional defects likely arise from reduced proteasome function due to UBR4 loss. Consistent with this model, muscle-specific inhibition of the proteasome by knockout of the Psmc4 subunit also leads to defects (although more severe than those induced by UBR4 loss) in muscle function in mice^52^. Moreover, proteasome function is required to maintain muscle cellular architecture also in *Drosophila*^53^. Altogether, these findings suggest that UBR4 and the ubiquitin–proteasome system are necessary for optimal muscle function during aging.

In parallel with a decline in protein quality control, we find that UBR4 loss induces an adaptive stress response based on upregulation of the ubiquitin ligase STUB1/CHIP, which promotes the degradation of misfolded proteins and protects from sarcopenia^17,18^. Therefore, boosting adaptive compensatory responses that specifically target protein quality control may represent a promising avenue for treating sarcopenia.

Taken together, this study identified indispensable components of muscle protein quality control, i.e., UBR4, HGS, STAM, and USP8. We propose that preserving muscle protein quality control may provide an effective means to preserve muscle function during aging and extend a healthy lifespan.

## Methods

### *Drosophila* husbandry

Flies were maintained at 25 °C with 60% humidity on 12-h light/dark cycles unless otherwise specified with standard cornmeal/soy flour/yeast fly food. For adult muscle-specific manipulation, heterozygous *Mhc-GAL4.F3-580* (BDSC#38464 without *Mhc-RFP*) female virgins were crossed to UAS lines to produce a mix of GAL4 and UAS-containing flies alongside isogenic controls devoid of GAL4. white RNAi (TRIP HMS00017) was used as a control RNAi line unless specified otherwise. The UAS lines were obtained from the Bloomington Drosophila Stock Center and Vienna Drosophila Resource Center and are listed in Supplementary Data 3.

### Mouse procedures

All mice were housed and handled in accordance with approved St. Jude Children’s Research Hospital Institutional Animal Care and Use Committee protocols and fed standard chow with 12-h light/dark cycles. UBR4 muscle-specific knockdown, knockout, controls, and muscle functional analyses were performed as follows^16^.

For RNAi knockdown, the tibialis anterior (TA) muscles of male C57BL6/J mice were electroporated with either UBR4 or NT siRNA (Dharmacon) by first anesthetizing mice with isoflurane, removing hair from the hind legs, and then injecting the tibialis anterior with 30 mL of 0.4 U/L Hyaluronidase using a 29G1/2 insulin syringe. After recovering for two hours, the mice were anesthetized again and 500 pmol of siRNAs in 50 mL of Dharmacon’s recommended siRNA resuspension buffer (60 mM KCl, 6 mM HEPES-pH 7.5, and 0.2 mM MgCl_2_) were injected into the tibialis anterior muscle (one leg injected with NT siRNAs and the contralateral leg with UBR4 siRNA) followed by electroporation by using a BTX ECM 830 apparatus with metal electrodes placed parallel to the tibia orientation. Specifically, 4 pulses at 200 V/cm with 20 ms length at 1 Hz were delivered, followed by another 4 pulses after the orientation of the electrodes was switched to perpendicular to the tibia. After 7 days from electroporation, mice were sacrificed and TA muscles dissected and immediately snap-frozen. Subsequently, the transcript levels, protein levels, and histological parameters of the tibialis anterior muscles were examined.

For knockout, UBR4 floxed mice were bred together with ACTA1-Cre mice^23^ to yield homozygous UBR4fl/fl alleles either with (UBR4 mKO) or without ACTA1-Cre (wild-type controls). At 3 months of age, mice received daily intraperitoneal injections of 1 mg/kg of tamoxifen for 5 days to induce Cre-mediated recombination (UBR4 mKO mice). The wild-type siblings were used as matched controls that were injected with tamoxifen but where no recombination occurred due to the lack of the Cre recombinase.

### Cell culture experiments

C2C12 cells obtained from the ATCC were maintained in standard conditions, i.e., DMEM containing 10% FBS in a 5% CO_2_ incubator at 37 °C. To induce differentiation, C2C12 cells were grown to near confluence in 10% FBS-containing growth medium and differentiated in 2% horse serum-containing DMEM. siRNAs were obtained from Dharmacon and transfected^16^. C2C12 myoblasts were transfected with 50 mM siRNAs targeting the specified gene or with non-targeting (NT) siRNAs, using a ratio of 2 mL Lipofectamine 2000 to 50 pmol of siRNA in OptiMEM. Cells were tested 3 days after transfection. For experiments with starvation (Dulbecco’s PBS), MG132 (50 µM), and chloroquine (10 µM) to assess protein flux the cells were treated for 3 h. For cycloheximide chase experiments. cells were incubated with cycloheximide (100 µg/mL) in Dulbecco’s PBS for the indicated time periods up to 6 h. When testing UBR4 knockdown with MG132, chloroquine, and cycloheximide, cells were pre-incubated with cycloheximide for 24 h before treatment with MG132 or chloroquine for 6 h. At the conclusion of treatments, cells were collected in NP40 lysis buffer with protease inhibitors on ice and combined with SDS-loading buffer for western blotting.

### Protein degradation and proteasomal activity assays

Protein degradation was measured by growing siRNA-transfected C2C12 cells to confluence, switching them to Dulbecco’s Phosphate buffered saline (DPBS), and measuring the tyrosine released into the DPBS over time^54^. Protein extracts confirmed there was no significant difference in starting total protein levels between controls and UBR4 siRNA prior to switching to DPBS. The DPBS solutions at the noted timepoints were removed and ¼ volume of trichloroacetic acid added then centrifuged at 4 °C and 12,000 × *g* to precipitate and remove proteins. Supernatants were removed and 40 µL added to a 96-well plate as well as 40 µL of tyrosine standards. 100 µL of 500 mM sodium carbonate was added to each well, followed by 20 µL of 0.5 N Folin and Ciocalteu’s phenol reagent (Sigma #F9252) in pure water. Plates were incubated for 30 min at 37 °C and then read at 660 nm to estimate the levels of free tyrosine on the basis of the standards.

The proteasomal activity was measured by using the Promega Proteasome-Glo 3-substrate cell-based assay system (Promega #G1180). Eight biological replicates of cultured cells with siRNA transfection were prepared as above in white 96-well plates, and the recommended manufacturer protocol was followed. Specifically, 100 µL of the three different substrate reagents were added individually to plates with control and UBR4 siRNAs in 100 µL of cell culture media. The plates were then incubated for 30 min at room temperature and the luminescence read on a Tecan Infinite 200 PRO plate reader.

### RNA-seq and qRT-PCR

Tissues were homogenized in Trizol and RNA extracted by isopropanol precipitation from the aqueous phase. RNA sequencing was performed from UBR4 mKO tibialis anterior muscle at 6 and 24 months of age^16^. RNA sequencing libraries for each sample were prepared with 1 μg total RNA using the Illumina TruSeq RNA Sample Prep v2 Kit per the manufacturer’s instructions, and sequencing was completed on the Illumina HiSeq 4000. The 100 bp paired-end reads were trimmed, filtered against quality (Phred-like Q20 or greater) and length (50 bp or longer), and aligned to a mouse reference sequence GRCm38 (UCSC mm10), using CLC Genomics Workbench v12.0.1 (Qiagen). For gene expression comparisons, we obtained the TPM (transcript per million) counts from the RNA-Seq Analysis tool. The differential gene expression analysis was performed using the non-parametric ANOVA using the Kruskal-Wallis and Dunn’s tests on log-transformed TPM between three replicates of experimental groups, implemented in Partek Genomics Suite v7.0 software (Partek Inc.). The data discussed in this publication have been deposited in the NCBI’s Gene Expression Omnibus and are accessible through GEO Series accession number G7.

For qRT-PCR, cDNAs were reverse transcribed from 500 ng total RNA. The oligonucleotide primers used for detection of gene expression are listed in Supplementary Data 4. qRT-PCR was performed using GAPDH, HPRT, and PPIA as a normalization for mouse samples whereas Tub84B served as a normalization for *Drosophila* samples^55^.

### *Drosophila* lifespan analysis and muscle function assays

The lifespan of flies was assessed by counting deaths when changing food typically every 4 days. RNAi lines targeting ubiquitin-related genes were tested at 29 °C.

Muscle functional assays were done as before^8^. In brief, flight capacity was assessed by releasing flies into a clear box and counting the number of flies that could fly and that could not (i.e., that dropped immediately downward) to derive the percentage of flies not flying.

Climbing speed was measured by taking videos of flies in their tubes after tapping them down to induce negative geotaxis. The videos were analyzed by using ImageJ to determine the distance traveled from the bottom of the food for each fly after a 2 s period from being tapped down, thus deriving the average speed (cm/s) for that period. A typical cohort of flies consisted of 50–200 flies for each genotype and the mean for each genotype is represented in the figures.

### Western blots and analysis of detergent-insoluble protein fractions

Detergent-soluble protein fractions were extracted by homogenizing tissues in NP40 cell lysis buffer (Invitrogen #FNN0021; 100 µL per ~5 mg of mouse muscle tissue, or 100 µL per 5 fly thoraces) using a bullet blender (NextAdvance) with zirconium 0.5-mm beads, and then centrifuging at 12,000 × *g* for 10 min to pellet insoluble material. The supernatant (detergent-soluble protein fraction) was removed and the protein concentration determined by using the Bio-Rad protein assay. Subsequently, equal protein quantities of detergent-soluble fractions were loaded into 4–20% gradient polyacrylamide gels (Bio-Rad) with SDS blue loading buffer. Insoluble pellets were washed three times with NP40 cell lysis buffer. Subsequently, 8 M urea and 1% SDS in PBS was added to solubilize the insoluble pellet (the specific volume added was determined based on the concentration of the corresponding detergent-soluble fraction), combined with SDS blue loading buffer, and loaded into polyacrylamide gels. The gels were transferred to PVDF membranes and blocked with either 10% skim milk powder or BSA in Tris-buffered saline with 0.1% Tween-20 (TBST) for 1 h. The membranes were then incubated with 1:1000 dilutions of primary antibodies in TBST (mouse monoclonal P4D1 anti-ubiquitin Cell Signaling #3936, rabbit polyclonal anti-Ref(2)P Abcam #178440 for *Drosophila* SQSTM1/p62, rabbit monoclonal anti-tubulin (11H10) Cell Signaling #2125, rabbit monoclonal anti-GFP (D5.1) Cell Signaling #2956, rabbit polyclonal anti-UBR4 Abcam #86738, rabbit polyclonal anti-LC3 Sigma #L7543, rabbit polyclonal anti-SQSTM1/p62 Cell Signaling #5114, and rabbit monoclonal anti-STUB1 (C3B6) Cell Signaling #2080) overnight at 4 °C, washed and incubated with either anti-mouse or anti-rabbit HRP-linked secondary antibodies (Cell Signaling #7076 and #7074 respectively) for 2 h. Membranes were washed and incubated with ECL (GE Healthcare) and film used to measure the signal. Adobe Photoshop was used to quantify band density and proteins were normalized to tubulin band density and presented as a ratio relative to controls. For analysis of Htt-72Q-GFP with antibodies against GFP, flies were homogenized directly into SDS-loading buffer containing 8 M urea to extract all proteins and aggregates of high-molecular weights (>250 kDa). Uncropped and unprocessed scans of the western blots are shown in the Source Data File.

### Immunostaining and laser scanning confocal microscopy

For immunostaining of *Drosophila* thoracic indirect flight muscles^56^, whole flies were frozen on glass slides with OCT in liquid nitrogen and then bisected longitudinally at the median plane to obtain hemithorax sections, similar to a previously described procedure to obtain transverse sections^57^. Subsequently, hemithorax sections were fixed with 4% paraformaldehyde and 0.1% Triton X-100 for 30 min, washed, and then incubated with 1:200 dilutions of primary and secondary antibodies. The primary antibodies used were mouse monoclonal FK2, anti-tubulin, rabbit polyclonal anti-Ref(2)P (Abcam #178440), and chicken anti-GFP (Aves #GFP-1010). Secondary antibodies used were AlexaFluor conjugated. Stained thoraces were mounted on glass slides by polyvinyl-alcohol with DABCO (Sigma) and #1.5 coverslips and imaged using a Nikon C2 confocal microscope. ImageJ was used to quantify the aggregate particles by thresholding.

For mouse sections, the muscles were bisected at the mid-belly, mounted onto tragacanth gum, and frozen in isopentane cooled by liquid nitrogen. 10 µm sections were cut on a cryostat and immunostained^16^. Unfixed slides holding the sections were incubated with blocking buffer (PBS with 1% BSA and 2% horse serum) for 1 h before incubation with primary antibodies against type IIA (SC-71) and IIB myosin heavy chain (BF-F3) and laminin a2 overnight at 4 °C. The sections were then washed and incubated with secondary antibodies for type 2A (anti-mouse IgG1 Alexa488), type 2B (anti-mouse IgM Alexa555), and laminin (anti-rat IgG Alexa633). The whole tibialis anterior section was imaged on a Nikon C2 confocal microscope with a ×10 objective and stitched to compile an overview of the muscle. The fiber types and sizes were analyzed with the Nikon Elements software using the inverse threshold of laminin a2 staining to determine myofiber boundaries. The myosin heavy chain staining was used to classify type IIB fibers (red), type IIA (green), and presumed IIX fibers (black) that were not stained for IIB or IIA. After myofibers were classified and parameters measured, the Feret’s minimal diameter was used as the measurement of myofiber size due to its accuracy in estimating the size of unevenly shaped or cut objects. For the analysis of soleus muscles, the BA-F8 antibody for type I myosin heavy chain was used with a corresponding secondary antibody for the IgG2b isotype. For the quantification of the number of myofibers, all fibers in the cross-sections of entire tibialis anterior muscles were counted based on the myofiber borders identified by laminin immunostaining. The size and number of myofibers were measured from the inverse images of laminin immunostaining (for identifying myofiber borders), excluding myofibers with diameters <5 and >100 µm. To categorize myofiber types, the intersections of the inverse images of laminin and myosin heavy chain-specific staining were used. These analyses were performed using the Nikon Elements software and the “Object count” function.

### Protein digestion and peptide isobaric labeling by tandem mass tags

For the preparation of tibialis anterior muscles for TMT-MS, muscle samples were ground into a fine powder using a mortar and pestle cooled by liquid nitrogen, and similar volumes of the powdered samples were used for subsequent analyses, which were performed with slight modifications to a previously published protocol^58^. Specifically, tissue samples were extracted in lysis buffer (50 mM HEPES, pH 8.5, 8 M urea, and 0.5% sodium deoxycholate), and protein concentration of the lysates was determined by Coomassie-stained short gels using bovine serum albumin (BSA) as a standard^59^. 100 µg of protein for each sample was digested with LysC (Wako) at an enzyme-to-substrate ratio of 1:100 (w/w) for 2 h in the presence of 1 mM DTT. Following this, the samples were diluted to a final 2 M Urea concentration with 50 mM HEPES (pH 8.5), and further digested with trypsin (Promega) at an enzyme-to-substrate ratio of 1:50 (w/w) for at least 3 h. The peptides were reduced by adding 1 mM DTT for 30 min at room temperature (RT) followed by alkylation with 10 mM iodoacetamide (IAA) for 30 min in the dark at RT. The unreacted IAA was quenched with 30 mM DTT for 30 min. Finally, the digestion was terminated and acidified by adding trifluoroacetic acid (TFA) to 1%, desalted using C18 cartridges (Harvard Apparatus), and dried by speed vac. The purified peptides were resuspended in 50 mM HEPES (pH 8.5) and labeled with 16-plex Tandem Mass Tag (TMT) reagents (ThermoScientific) following the manufacturer’s recommendations.

### Two-dimensional HPLC and mass spectrometry

The TMT-labeled samples were mixed equally, desalted, and fractionated on an offline HPLC (Agilent 1220) using basic pH reverse-phase liquid chromatography (pH 8.0, XBridge C18 column, 4.6 mm × 25 cm, 3.5 μm particle size, Waters). The fractions were dried and resuspended in 5% formic acid and analyzed by acidic pH reverse phase LC-MS/MS analysis. The peptide samples were loaded on a nanoscale capillary reverse phase C18 column (New objective, 75 um ID × ~25 cm, 1.9 μm C18 resin from Dr. Maisch GmbH) by an HPLC system (Thermo Ultimate 3000) and eluted by a 60-min gradient. The eluted peptides were ionized by electrospray ionization and detected by an inline Orbitrap Fusion mass spectrometer (ThermoScientific). The mass spectrometer is operated in data-dependent mode with a survey scan in Orbitrap (60,000 resolution, 1 × 10^6^ AGC target and 50 ms maximal ion time) and MS/MS high-resolution scans (60,000 resolution, 2 × 10^5^ AGC target, 120 ms maximal ion time, 32 HCD normalized collision energy, 1 *m*/*z* isolation window, and 15 s dynamic exclusion).

### MS data analysis

The MS/MS raw files were processed by the tag-based hybrid search engine, JUMP, which showed better sensitivity and specificity than commercial packages (e.g., Proteome Discoverer)^60^. The raw data were searched against the UniProt mouse database concatenated with a reversed decoy database for evaluating false discovery rate. Searches were performed using a 15-ppm mass tolerance of both precursor and product ions, fully tryptic restriction with two maximal missed cleavages, three maximal modification sites, and the assignment of *a*, *b*, and *y* ions. TMT tags on Lys and N-termini (+304.20715 Da) were used for static modifications and Met oxidation (+15.99492 Da) was considered as a dynamic modification. Matched MS/MS spectra were filtered by mass accuracy and matching scores to reduce protein false discovery rate to ~1%. Proteins were quantified by summing reporter ion intensities across all matched PSMs using the JUMP software suite^61^.

### Measurements of muscle force production

The in situ contractile function of the tibialis anterior muscle was measured^16^. Mice were deeply anesthetized via i.p. injection of ketamine–xylazine (80 and 10 mg/kg) and monitored throughout the experiment. The distal tendon of the tibialis anterior was carefully dissected and individually tied with a 5.0 braided silk suture. The sciatic nerve was exposed and all branches were cut except for the common peroneal nerve. The foot was secured to a platform and the knee immobilized using a stainless-steel pin. The body temperature was monitored and maintained at 37 °C. The suture from the tendon was individually attached to the lever arm of a 305B dual-mode servomotor transducer (Aurora Scientific, Ontario, Canada). Muscle contractions were then elicited by stimulating the distal part of the sciatic via bipolar electrodes, using supramaximal square-wave pulses of 0.2 ms (701A stimulator; Aurora Scientific). Data acquisition and control of the servomotor were conducted using a Lab-View-based DMC program (version 5.420; Aurora Scientific). Optimal muscle length (Lo) was determined by incrementally stretching the muscle until the maximum isometric twitch force was achieved. Three maximum isometric tetanic forces (Po) were acquired using a train of 150 Hz supramaximal electrical pulses for 500 ms at the optimal length in the muscles and the highest Po was recorded. A 2-min resting period was allowed between each tetanic contraction. Lo was measured using digital calipers. The relative force (*N*/*g*) was normalized by dividing Po by the muscle weight.

### Statistics and reproducibility

The unpaired two-tailed Student’s *t*-test was used to compare the means of two independent groups to each other. One-way ANOVA with Tukey’s post hoc test was used for multiple comparisons of more than two groups of normally distributed data. For analysis of lifespan, the OASIS2 webtool^62^ was used for log-rank comparisons of mean lifespan. The *n* for each experiment can be found in the figures and legends and represents independently generated samples for all experiments, including cell populations/wells for in vitro assays, and batches of flies and samples from individual mice for in vivo experiments. For western blotting, two independent groups of biological replicates were tested and produced similar results consistent with the conclusions with one set represented in figures. Bar graphs present the mean ± SEM. Throughout the figures where *P*-value is not annotated, asterisks indicate the significance of the *P*-value: *P < 0.05. A significant result was defined as *P* < 0.05. Statistical analyses were done with Excel and GraphPad Prism. Venn diagrams were created with Venny^63^ (https://bioinfogp.cnb.csic.es/tools/venny/).

### Reporting summary

Further information on research design is available in the Nature Research Reporting Summary linked to this article.

## Supplementary information

Supplementary Information

Supplementary Data 1

Supplementary Data 2

Supplementary Data 3

Supplementary Data 4

Supplementary Data 5

Supplementary Data 6

Supplementary Data 7

Reporting Summary

Description of Additional Supplementary Files

## Data Availability

All data supporting the findings of this study are available within the paper and the Supplementary information, including Supplementary Data 1–7 and the Source Data file. The RNA-seq data generated from this study has been deposited in the NCBI’s Gene Expression Omnibus and is publicly accessible through GEO Series accession number GSE149637. Source data are provided with this paper.

## Source data

Source data

## References

[1] Johnston AJ (2015). Targeting of Fn14 prevents cancer-induced cachexia and prolongs survival. Cell.

[2] Tisdale MJ (2010). Reversing cachexia. Cell.

[3] Zhou X (2010). Reversal of cancer cachexia and muscle wasting by ActRIIB antagonism leads to prolonged survival. Cell.

[4] Bonaldo P, Sandri M (2013). Cellular and molecular mechanisms of muscle atrophy. Dis. Models Mech..

[5] Reid MB, Judge AR, Bodine SC (2014). CrossTalk opposing view: the dominant mechanism causing disuse muscle atrophy is proteolysis. J. Physiol..

[6] Demontis F, Perrimon N (2010). FOXO/4E-BP signaling in Drosophila muscles regulates organism-wide proteostasis during aging. Cell.

[7] Demontis F, Piccirillo R, Goldberg AL, Perrimon N (2013). The influence of skeletal muscle on systemic aging and lifespan. Aging Cell.

[8] Hunt LC (2019). Circadian gene variants and the skeletal muscle circadian clock contribute to the evolutionary divergence in longevity across Drosophila populations. Genome Res..

[9] Jiao J, Demontis F (2017). Skeletal muscle autophagy and its role in sarcopenia and organismal aging. Curr. Opin. Pharm..

[10] Carnio S (2014). Autophagy impairment in muscle induces neuromuscular junction degeneration and precocious aging. Cell Rep..

[11] Castets P (2013). Sustained activation of mTORC1 in skeletal muscle inhibits constitutive and starvation-induced autophagy and causes a severe, late-onset myopathy. Cell Metab..

[12] Demontis F, Piccirillo R, Goldberg AL, Perrimon N (2013). Mechanisms of skeletal muscle aging: insights from Drosophila and mammalian models. Dis. Models Mech..

[13] Masiero E (2009). Autophagy is required to maintain muscle mass. Cell Metab..

[14] Sandri M (2013). Signalling pathways regulating muscle mass in ageing skeletal muscle: the role of the IGF1-Akt-mTOR-FoxO pathway. Biogerontology.

[15] Hwee DT, Baehr LM, Philp A, Baar K, Bodine SC (2014). Maintenance of muscle mass and load-induced growth in Muscle RING Finger 1 null mice with age. Aging Cell.

[16] Hunt LC (2019). A key role for the ubiquitin ligase UBR4 in myofiber hypertrophy in Drosophila and mice. Cell Rep..

[17] Arndt, V. et al. Chaperone-assisted selective autophagy is essential for muscle maintenance. *Curr. Biol.* **20**, 143–148 (2010). 10.1016/j.cub.2009.11.022 20060297

[18] Min JN (2008). CHIP deficiency decreases longevity, with accelerated aging phenotypes accompanied by altered protein quality control. Mol. Cell. Biol..

[19] Kumar V (2009). Age-related differences in the dose-response relationship of muscle protein synthesis to resistance exercise in young and old men. J. Physiol..

[20] Brook MS (2016). Synchronous deficits in cumulative muscle protein synthesis and ribosomal biogenesis underlie age-related anabolic resistance to exercise in humans. J. Physiol..

[21] Hodson N, West DWD, Philp A, Burd NA, Moore DR (2019). Molecular regulation of human skeletal muscle protein synthesis in response to exercise and nutrients: a compass for overcoming age-related anabolic resistance. Am. J. Physiol. Cell Physiol..

[22] van Dijk M (2017). Sarcopenia in older mice is characterized by a decreased anabolic response to a protein meal. Arch. Gerontol. Geriatr..

[23] McCarthy JJ, Srikuea R, Kirby TJ, Peterson CA, Esser KA (2012). Inducible Cre transgenic mouse strain for skeletal muscle-specific gene targeting. Skelet. Muscle.

[24] Piccirillo R, Demontis F, Perrimon N, Goldberg AL (2014). Mechanisms of muscle growth and atrophy in mammals and Drosophila. Dev. Dyn..

[25] Osvaldo D (2011). Expression and regulation of excitation-contraction coupling proteins in aging skeletal muscle. Curr. Aging Sci..

[26] Davey, J. R. et al. Integrated expression analysis of muscle hypertrophy identifies Asb2 as a negative regulator of muscle mass. *JCI Insight* **1**, 10.1172/jci.insight.85477 (2016). 10.1172/jci.insight.85477 PMC4863241 27182554

[27] Qian SB, McDonough H, Boellmann F, Cyr DM, Patterson C (2006). CHIP-mediated stress recovery by sequential ubiquitination of substrates and Hsp70. Nature.

[28] Besche HC, Haas W, Gygi SP, Goldberg AL (2009). Isolation of mammalian 26S proteasomes and p97/VCP complexes using the ubiquitin-like domain from HHR23B reveals novel proteasome-associated proteins. Biochemistry.

[29] Besche HC (2014). Autoubiquitination of the 26S proteasome on Rpn13 regulates breakdown of ubiquitin conjugates. EMBO J..

[30] Tasaki T (2013). UBR box N-recognin-4 (UBR4), an N-recognin of the N-end rule pathway, and its role in yolk sac vascular development and autophagy. Proc. Natl Acad. Sci. USA.

[31] Gajewski KM, Schulz RA (2010). CF2 represses Actin 88F gene expression and maintains filament balance during indirect flight muscle development in Drosophila. PLoS ONE.

[32] Vietri M, Radulovic M, Stenmark H (2020). The many functions of ESCRTs. Nat. Rev. Mol. Cell Biol..

[33] Kim ST (2018). The N-recognin UBR4 of the N-end rule pathway is required for neurogenesis and homeostasis of cell surface proteins. PLoS ONE.

[34] Kim ST (2018). The N-recognin UBR4 of the N-end rule pathway is targeted to and required for the biogenesis of the early endosome. J. Cell Sci..

[35] Wright MH, Berlin I, Nash PD (2011). Regulation of endocytic sorting by ESCRT–DUB-mediated deubiquitination. Cell Biochem. Biophys..

[36] Albin RL (1993). Antagonistic pleiotropy, mutation accumulation, and human genetic disease. Genetica.

[37] Williams PD, Day T (2003). Antagonistic pleiotropy, mortality source interactions, and the evolutionary theory of senescence. Evolution.

[38] Joseph, G. A. et al. Partial inhibition of mTORC1 in aged rats counteracts the decline in muscle mass and reverses molecular signaling associated with sarcopenia. *Mol. Cell Biol.* **39**, 10.1128/MCB.00141-19 (2019). 10.1128/MCB.00141-19 PMC6751631 31308131

[39] Amthor H (2007). Lack of myostatin results in excessive muscle growth but impaired force generation. Proc. Natl Acad. Sci. USA.

[40] Tinklenberg JA (2018). Myostatin inhibition using mRK35 produces skeletal muscle growth and tubular aggregate formation in wild type and TgACTA1D286G nemaline myopathy mice. Hum. Mol. Genet..

[41] Musarò A (2001). Localized Igf-1 transgene expression sustains hypertrophy and regeneration in senescent skeletal muscle. Nat. Genet..

[42] Barton-Davis ER, Shoturma DI, Musaro A, Rosenthal N, Sweeney HL (1998). Viral mediated expression of insulin-like growth factor I blocks the aging-related loss of skeletal muscle function. Proc. Natl Acad. Sci. USA.

[43] Ascenzi F (2019). Effects of IGF-1 isoforms on muscle growth and sarcopenia. Aging Cell.

[44] Milan G (2015). Regulation of autophagy and the ubiquitin-proteasome system by the FoxO transcriptional network during muscle atrophy. Nat. Commun..

[45] Demontis F, Perrimon N (2009). Integration of Insulin receptor/Foxo signaling and dMyc activity during muscle growth regulates body size in Drosophila. Development.

[46] Demontis F, Patel VK, Swindell WR, Perrimon N (2014). Intertissue control of the nucleolus via a myokine-dependent longevity pathway. Cell Rep..

[47] Goldberg AL (1969). Protein turnover in skeletal muscle II. Effects of denervation and cortisone on protein catabolism in skeletal muscle. J. Biol. Chem..

[48] Rinschen MM (2016). The ubiquitin ligase Ubr4 controls stability of podocin/MEC-2 supercomplexes. Hum. Mol. Genet..

[49] Lin R (2013). Acetylation stabilizes ATP-citrate lyase to promote lipid biosynthesis and tumor growth. Mol. Cell.

[50] Yang HB (2015). Acetylation of MAT IIalpha represses tumour cell growth and is decreased in human hepatocellular cancer. Nat. Commun..

[51] Yau RG (2017). Assembly and function of heterotypic ubiquitin chains in cell-cycle and protein quality control. Cell.

[52] Kitajima Y (2014). Proteasome dysfunction induces muscle growth defects and protein aggregation. J. Cell Sci..

[53] Haas KF, Woodruff E, Broadie K (2007). Proteasome function is required to maintain muscle cellular architecture. Biol. Cell.

[54] Cupp-Enyard, C. Sigma’s non-specific protease activity assay—casein as a substrate. *J. Vis. Exp.*10.3791/899 (2008). 10.3791/899 PMC2872977 19066538

[55] Hunt LC (2015). The glucose-sensing transcription factor MLX promotes myogenesis via myokine signaling. Genes Dev..

[56] Hunt LC, Demontis F (2013). Whole-mount immunostaining of Drosophila skeletal muscle. Nat. Protoc..

[57] Rai M, Nongthomba U (2013). Effect of myonuclear number and mitochondrial fusion on Drosophila indirect flight muscle organization and size. Exp. Cell Res..

[58] Bai B (2017). Deep profiling of proteome and phosphoproteome by isobaric labeling, extensive liquid chromatography, and mass spectrometry. Methods Enzymol..

[59] Xu P, Duong DM, Peng J (2009). Systematical optimization of reverse-phase chromatography for shotgun proteomics. J. Proteome Res..

[60] Wang X (2014). JUMP: a tag-based database search tool for peptide identification with high sensitivity and accuracy. Mol. Cell. Proteom..

[61] Pagala VR (2015). Quantitative protein analysis by mass spectrometry. Methods Mol. Biol..

[62] Han, S. K. et al. OASIS 2: online application for survival analysis 2 with features for the analysis of maximal lifespan and healthspan in aging research. *Oncotarget* **7**, 56147–56152 (2016). 10.18632/oncotarget.11269 PMC5302902 27528229

[63] Oliveros, J. C. VENNY. An interactive tool for comparing lists with Venn diagrams. http://bioinfogp.cnb.csic.es/tools/venny/index.html (2007).

