# Supplementary material for: Antagonistic control of myofiber size and muscle protein quality control by the ubiquitin ligase UBR4 during aging
Source: Nat Commun. 2021 Mar 3;12:1418. doi: 10.1038/s41467-021-21738-8 (PMC7930053; doi:10.1038/s41467-021-21738-8)
Supplement: Supplementary file 1 — Supplementary Information [file 41467_2021_21738_MOESM1_ESM.pdf]

SUPPLEMENTARY INFORMATION

Antagonistic control of myofiber size and muscle protein quality control by the ubiquitin ligase UBR4 during aging

Liam C. Hunt, Bronwen Schadeberg, Jared Stover, Benard Haugen, Vishwajeeth Pagala, Yong-Dong Wang, Jason Puglise, Elisabeth R. Barton, Junmin Peng, Fabio Demontis

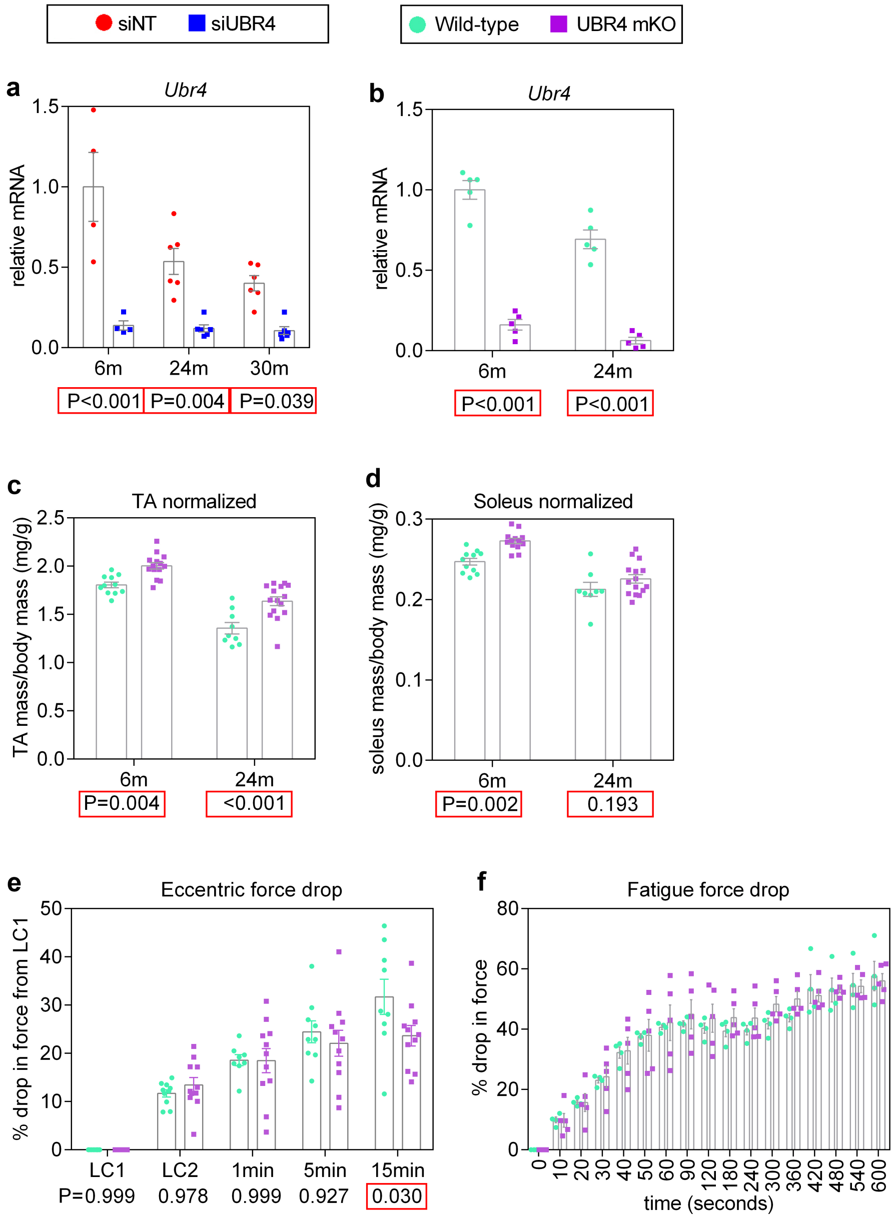

Supplementary Figure 1. Analysis of additional outcomes resulting from UBR4 knockout on mouse skeletal muscle mass and function.

a qPCR-verified knockdown of UBR4 mRNA obtained by siRNA injection and electroporation into tibialis anterior muscles at 6, 24, and 30 months of age (respectively n=4, n=6, and n=6/condition), **b** and by Cre-lox mediated muscle-specific knockout in the tibialis anterior muscles of mice (n=5).

c The masses of the tibialis anterior muscle **d** and soleus muscle normalized to body mass demonstrate a significant drop in the proportion of lean mass with age. Loss of UBR4 in young age increases the proportional mass for both muscles and for the tibialis anterior muscle in old age. In c-d, n=11 (6-month-old WT), n=13 (6-month-old UBR4 KO), n=9 (24-month-old WT), and n=14 (24-month-old UBR4 KO) muscles from independent mice.

e Following eccentric contractions, the drop in force due to the damaging lengthening contractions are reduced with UBR4 mKO; n=9 (WT) and n=10 (UBR4 KO) muscles from independent mice.

f However, the drop in force due to muscle fatigue is not significantly altered, suggesting that UBR4 mKO muscles may be better protected from damage induced by eccentric contraction; n=4 (WT) and n=5 (UBR4 KO) muscles from independent mice.

Data are presented as mean ± SEM. Statistics were calculated by using two-way ANOVA with Tukey’s post hoc test with adjustment for multiple comparisons.

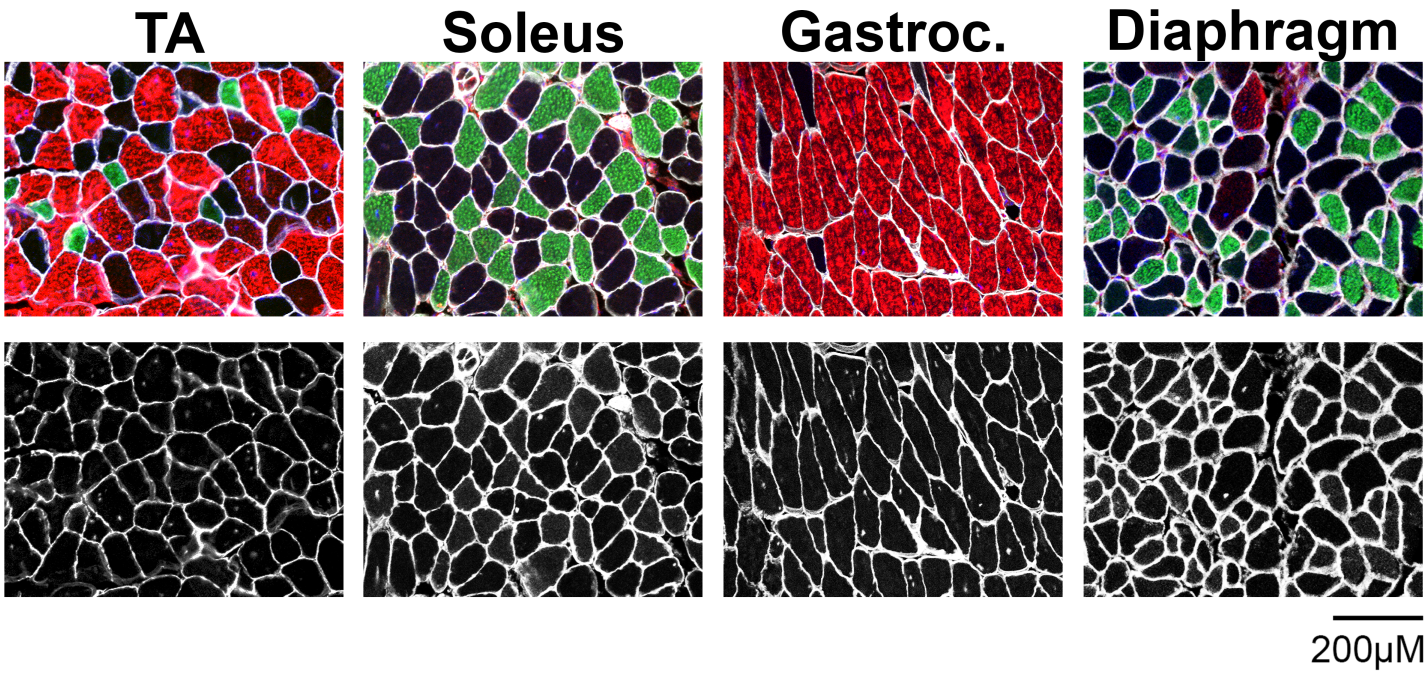

Supplementary Figure 2. Knockout of UBR4 affects skeletal muscles throughout the body in mice. In addition to the tibialis anterior (TA) muscle, other skeletal muscles display a similar phenotype in response to UBR4 mKO in mice: centralized nuclei, identified by DAPI staining (white), are present in myofibers of the soleus, gastrocnemius, and diaphragm muscles of UBR4 mKO mice but not in myofibers of muscles from wild-type controls (not shown). These centralized nuclei, which are indicative of myofiber degeneration and induction of compensatory regeneration, are most often present in type 2B myofibers which are more numerous in the TA and gastrocnemius in comparison to the diaphragm. Immunostaining for laminin (white) delineates myofiber boundaries whereas immunostaining for type 2A (green) and type 2B (red) myosin heavy chain identifies type 2A and type 2B myofibers. These findings suggest that knockout of UBR4 affects skeletal muscles throughout the body.

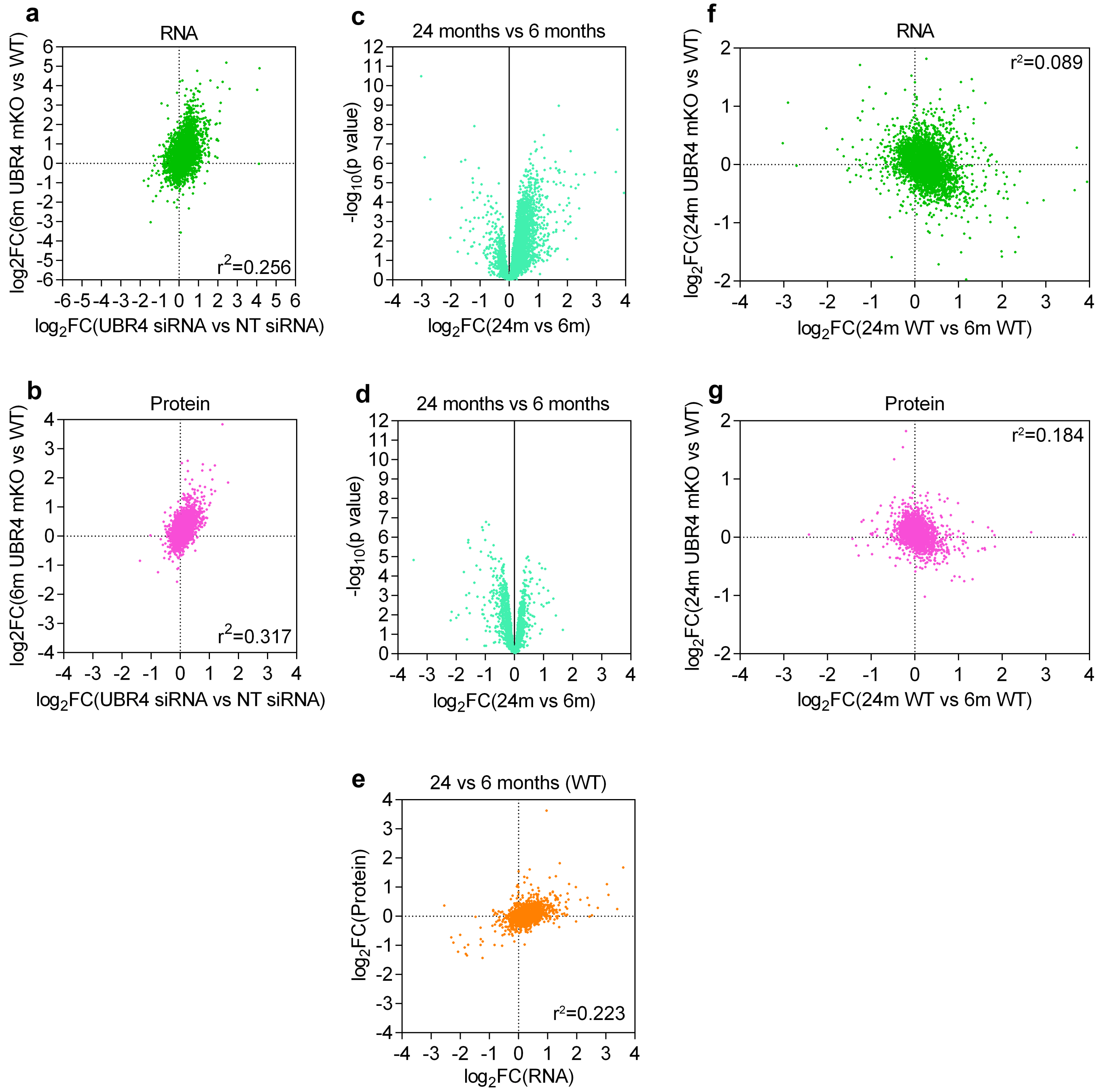

Supplementary Figure 3. Comparison of mRNA and protein changes induced by UBR4 mKO during aging in mice.

a-b In comparison to siRNA-mediated loss of UBR4, UBR4 muscle-specific knockout induces similar changes (albeit greater in magnitude) in mRNA (**a**) and protein (**b**) levels in the tibialis anterior muscle of young mice, skewing the linear relationship towards the vertical axis (UBR4 mKO log_2 fold changes). **c-d** Age-related changes in mRNA (**c**) and protein (**d**) levels are found when comparing 24-month to 6-month-old wild-type muscles. **e** There is a reasonable correlation between transcriptional and translational changes, although there is a skewing toward protein changes that do not correspond to mRNA changes. **f-g** Comparison of mRNA (**f**) and protein (**g**) changes caused by UBR4 mKO in old age versus changes caused by aging indicates that some mRNA/protein changes caused by loss of UBR4 may oppose aging.

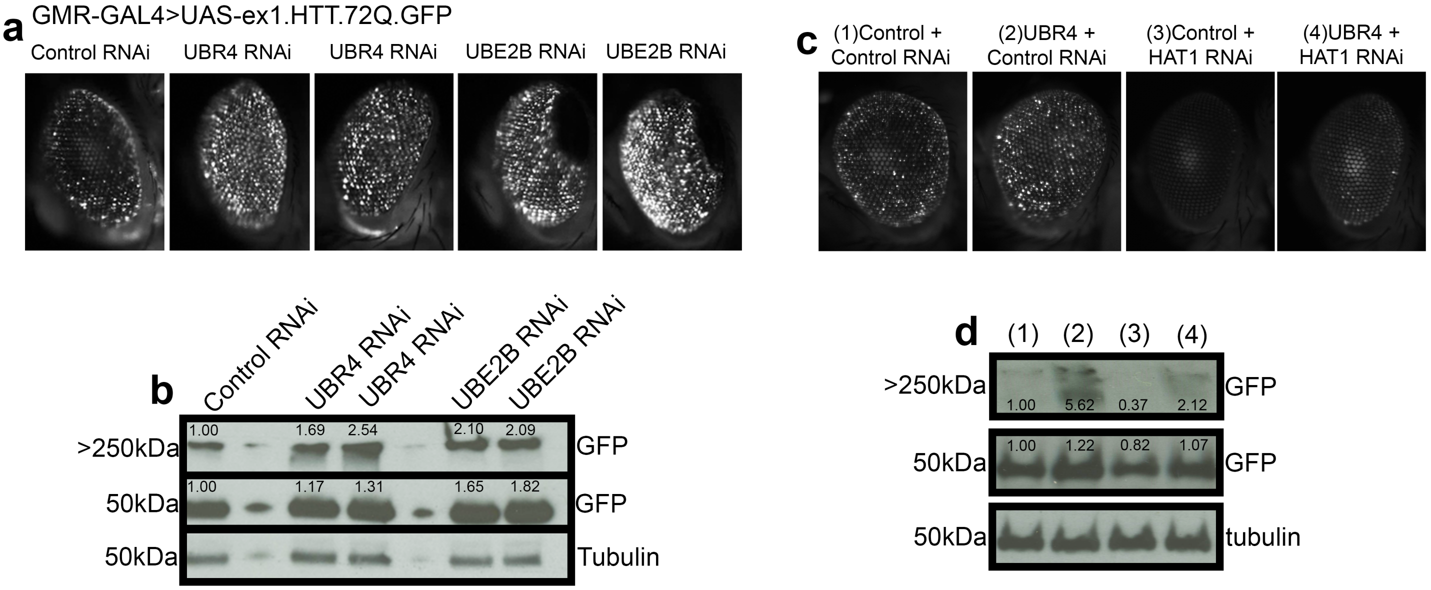

Supplementary Figure 4. The UBR4 pathway regulates protein quality control in *Drosophila* retinas with pathogenic huntingtin-polyQ.

In addition to regulating muscle protein quality control, the UBR4 pathway (UBR4/UBE2B/HAT1) also regulates proteostasis in *Drosophila* retinas with pathogenic huntingtin-polyQ. **a** *Drosophila* eyes of flies with knockdown of UBR4 and of UBE2B show increased Htt72Q-GFP aggregation (as estimated based on the increase in Htt72Q-GFP fluorescence), **b** which correspondingly increases the high molecular weight aggregates (>250 kDa) detected by western blot. **c-d** Epistasis shows that HAT1 is necessary for the increased Htt72Q-GFP aggregation induced by loss of UBR4. Specifically, Htt72Q-GFP aggregation is substantially reduced when UBR4 RNAi is combined with HAT1 RNAi, compared to a control RNAi targeting luciferase, as estimated based on **c** the Htt72Q-GFP fluorescence and **d** by western blot.

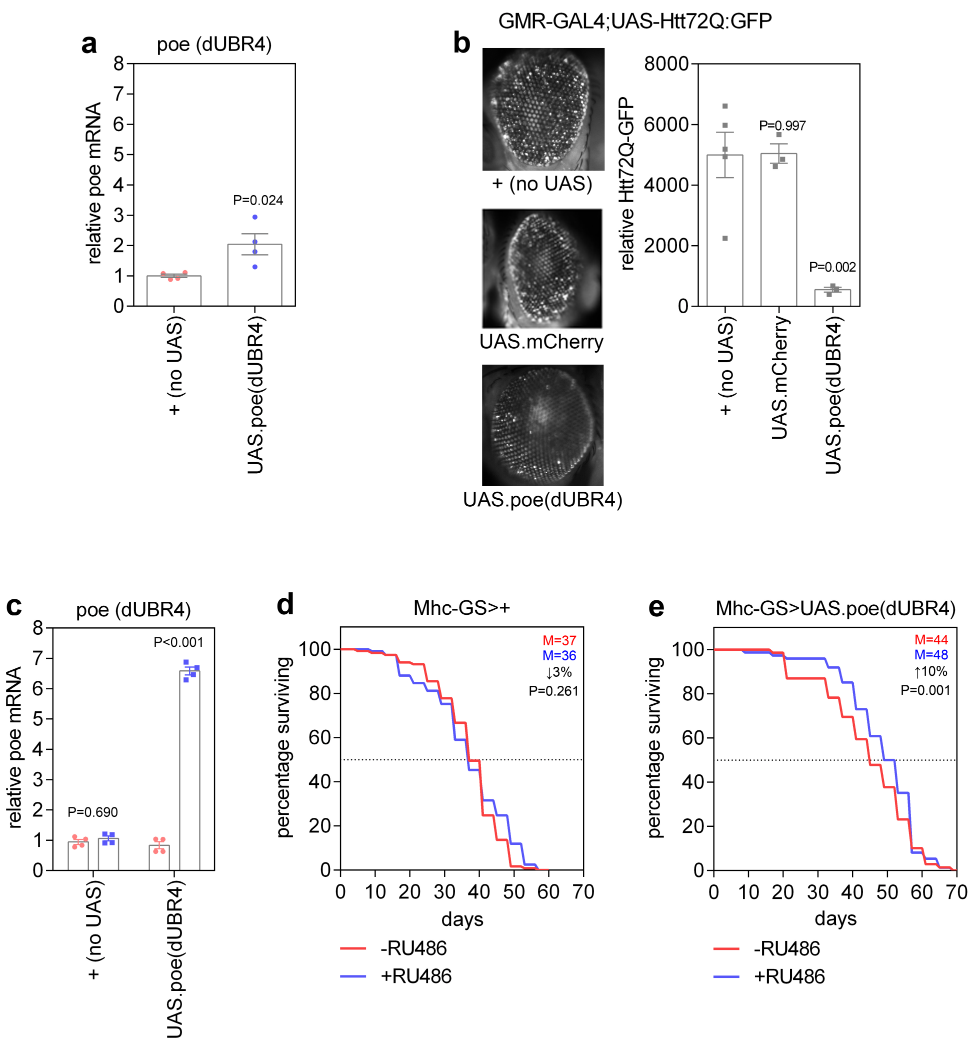

Supplementary Figure 5. *UBR4* overexpression improves protein quality control in the *Drosophila* retina and extends lifespan.

a Overexpression of *Drosophila UBR4* (*poe*) by using a P-element UAS insertion upstream of its open-reading frame. The UAS-poe(UBR4) line here used drives only mild *UBR4* overexpression (approximately 2-fold increase in mRNA above endogenous levels with *Mhc-Gal4*), as determined by qRT-PCR. Shown is the mean and SEM, with n=4. Statistical analysis was done with the two-tailed Student's t-test.

b *UBR4* overexpression reduces aggregates of GFP-tagged huntingtin-polyQ in the retina, as visualized by epifluorescence microscopy, compared to controls. Shown is the mean and SEM, with n=≥3. Statistical analysis was done with one-way ANOVA and Dunnett's post hoc test for multiple comparisons.

c UAS-poe(UBR4) drives stronger *UBR4* overexpression (approximately 7-fold increase in mRNA above endogenous levels) when using an inducible GeneSwitch driver in adult skeletal muscle (*Mhc-GS-Gal4*) after induction with RU486 (100 μM), as determined by qPCR. Shown is the mean and SEM, with n=4. Statistical analysis was done with two-way ANOVA and Tukey's post hoc test for multiple comparisons.

d RU486 treatment alone does not influence lifespan as demonstrated by comparing -RU486 to +RU486 conditions with no transgene expression (+) and *Mhc-GS-Gal4* (n=117/group).

e *UBR4* overexpression with *Mhc-GS-Gal4* increases the median lifespan compared to uninduced controls, suggesting that gain of function of UBR4 contributes to increase survival during aging (n[-RU486]=74 and n[+RU486]=69). In d-e, log-rank tests were performed to compare mean lifespans.

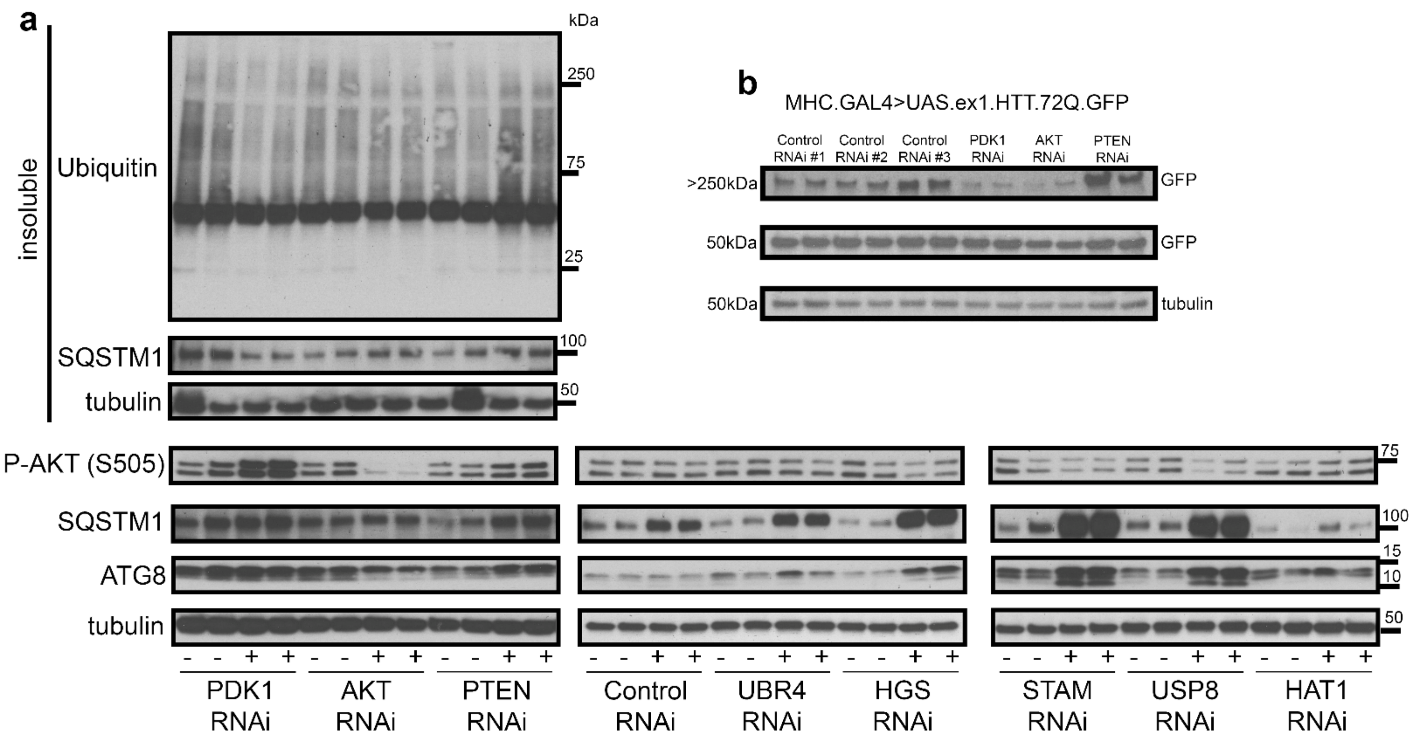

Supplementary Figure 6. Modulation of hypertrophic signaling by tested ubiquitin-related genes occurs without modulation of AKT activity.

a Consistent with a role in proteostasis for growth-regulating pathways, inhibition of PI3K-AKT signaling via RNAi for PDK1 and AKT reduces protein aggregation in muscle, as indicated by lower levels of ubiquitin and SQSTM1/Ref(2)P found in detergent-insoluble fractions. Conversely, PTEN RNAi (an inhibitor of PI3K) increases ubiquitin and SQSTM1 insoluble protein levels. Consistent with their known roles in modulating AKT activity, RNAi for AKT and PTEN elicited the expected reduction and increase in AKT phosphorylation, respectively. On the other hand, ubiquitin-related genes that modulate growth (i.e. UBR4, HGS, STAM, USP8 and HAT1) did not affect AKT phosphorylation although they impact proteostasis, as shown in Fig. 7 and herein (as demonstrated by the increased SQSTM1 levels found in detergent-insoluble fractions). Thus, modulation of growth and proteostasis by these ubiquitin-related genes does not seem to occur via PI3K-AKT signaling in this system.

b PI3K-AKT signaling also influence Htt72Q-GFP aggregation. Specifically, inhibition of this pathway (via PDK1/AKT RNAi) reduces high molecular weight aggregates of Htt72Q-GFP whereas pathway activation (via PTEN RNAi) increases aggregation.

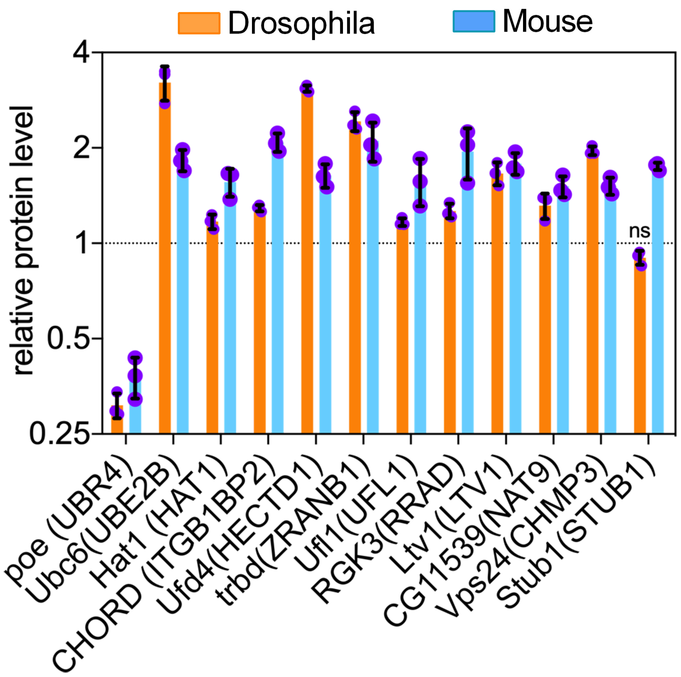

Supplemental Figure 7. Comparison of proteins modulated by UBR4 loss in *Drosophila* and mouse skeletal muscle. Loss of UBR4 induces overall similar changes in *Drosophila* and mouse skeletal muscles. For example, proteins previously shown to be direct interactors of UBR4, such as Ubc6/UBE2B and HAT1, are similarly upregulated by UBR4 loss in *Drosophila* and mouse muscles. However, a notable exception is STUB1/CHIP, which is upregulated in response to UBR4 loss in mouse but not in *Drosophila* muscle. This finding suggests that mouse skeletal muscle can at least in part compensate for decline in protein quality due to UBR4 loss via adaptive STUB1/CHIP upregulation, whereas this does not occur in *Drosophila*. All the proteins here indicated were significantly regulated by UBR4 RNAi in both *Drosophila* and mouse skeletal muscle ($P<0.05$), with the exception of STUB1/CHIP in *Drosophila* muscle; n=3 biological replicates each representing independent muscles from separate animals (for mice) or pools of 50 thoraces (for *Drosophila*). Data are presented as mean ± SEM. Statistical analysis was performed by one-way ANOVA with Benjamini-Hochberg false discovery testing for multiple comparisons.

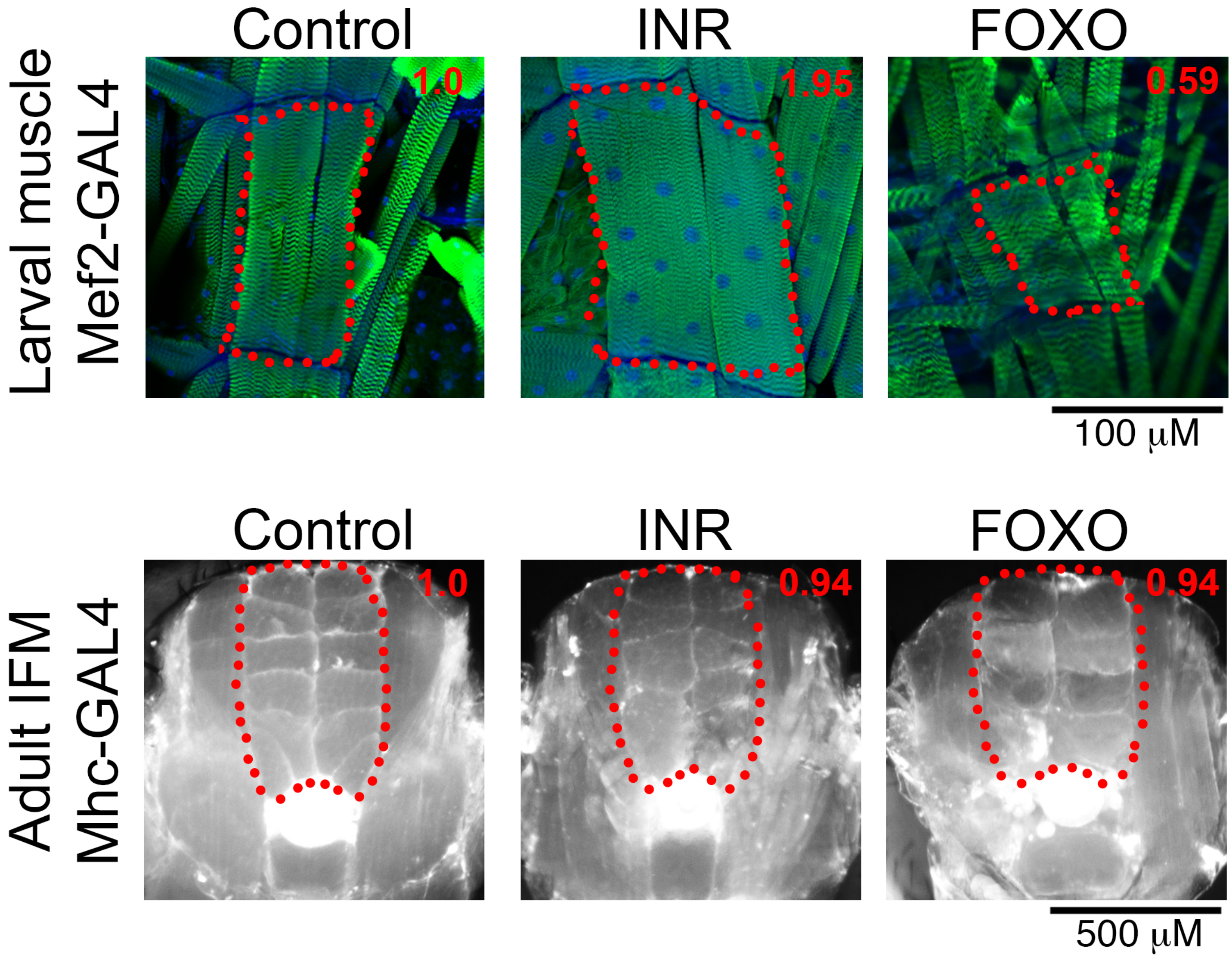

Supplementary Figure 8. Insulin receptor and FoxO modulate myofiber size in larval body wall muscles but not in indirect flight muscles of adult *Drosophila*.

Overexpression of *insulin receptor* (*INR*) and the transcription factor *FOXO* in body wall muscles of *Drosophila* larvae by using the *Mef2-GAL4* driver (top row), and in the indirect flight muscles (IFMs) of adult *Drosophila* by using a *Mhc-Gal4* line that does not drive meaningful expression during development (bottom row). During larval muscle growth, *INR* and *FOXO* overexpression induces myofiber hypertrophy and atrophy respectively (top row), as indicated by the analysis of VL3 and VL4 muscles (circled in red), each composed by a single myofiber. However, *INR* and *FOXO* overexpression with *Mhc-Gal4* does not change the size of myofibers that compose the IFMs of adult *Drosophila* (bottom row). The relative muscle area is indicated in red for each genotype. On this basis, we conclude that myofiber size is plastic and can be modulated by altering insulin signaling during developmental muscle growth but not in indirect flight muscle of adult flies. Specifically, there are no changes in myofiber size and muscle mass in the adult even with interventions that regulate protein quality control in adult IFMs and that modulate myofiber size developmentally. However, it is possible that the skeletal muscle cross-sectional area of adult *Drosophila* is affected in muscle pathologies that cause degeneration.
