# Supplementary material for: Antagonistic control of myofiber size and muscle protein quality control by the ubiquitin ligase UBR4 during aging
Source: Nat Commun. 2021 Mar 3;12:1418. doi: 10.1038/s41467-021-21738-8 (PMC7930053; doi:10.1038/s41467-021-21738-8)
Supplement: Supplementary file 9 — Reporting Summary [file 41467_2021_21738_MOESM9_ESM.pdf]

nature research

Corresponding author(s): Fabio Demontis

Last updated by author(s): 7 January 2021

Reporting Summary

Nature Research wishes to improve the reproducibility of the work that we publish. This form provides structure for consistency and transparency in reporting. For further information on Nature Research policies, see Authors & Referees and the Editorial Policy Checklist .

Statistics

For all statistical analyses, confirm that the following items are present in the figure legend, table legend, main text, or Methods section.

n/a	Confirmed	
☐	☒	The exact sample size (*n*) for each experimental group/condition, given as a discrete number and unit of measurement
☐	☒	A statement on whether measurements were taken from distinct samples or whether the same sample was measured repeatedly
☐	☒	The statistical test(s) used AND whether they are one- or two-sided<br>*Only common tests should be described solely by name; describe more complex techniques in the Methods section.*
☐	☒	A description of all covariates tested
☐	☒	A description of any assumptions or corrections, such as tests of normality and adjustment for multiple comparisons
☐	☒	A full description of the statistical parameters including central tendency (e.g. means) or other basic estimates (e.g. regression coefficient) AND variation (e.g. standard deviation) or associated estimates of uncertainty (e.g. confidence intervals)
☐	☒	For null hypothesis testing, the test statistic (e.g. *F*, *t*, *r*) with confidence intervals, effect sizes, degrees of freedom and *P* value noted<br>*Give P values as exact values whenever suitable.*
☒	☐	For Bayesian analysis, information on the choice of priors and Markov chain Monte Carlo settings
☒	☐	For hierarchical and complex designs, identification of the appropriate level for tests and full reporting of outcomes
☒	☐	Estimates of effect sizes (e.g. Cohen's *d*, Pearson's *r*), indicating how they were calculated

Our web collection on statistics for biologists contains articles on many of the points above.

Software and code

Policy information about availability of computer code

Data collection	Excel (version 15.30 or earlier), GraphPad (version 6.0b).
Data analysis	RNA-seq: RNA sequencing libraries for each sample were prepared with 1 µg total RNA using the Illumina TruSeq RNA Sample Prep v2 Kit per the manufacturer's instructions, and sequencing was completed on the Illumina HiSeq 4000. The 100 bp paired-end reads were trimmed, filtered against quality (Phred-like Q20 or greater) and length (50 bp or longer), and aligned to a mouse reference sequence GRCm38 (UCSC mm10), using CLC Genomics Workbench v12.0.1 (Qiagen). For gene expression comparisons, we obtained the TPM (transcript per million) counts from the RNA-Seq Analysis tool. The differential gene expression analysis was performed using the non-parametric ANOVA using the Kruskal-Wallis and Dunn's tests on log-transformed TPM between three replicates of experimental groups, implemented in Partek Genomics Suite v7.0 software (Partek Inc.).<br><br>TMT Mass spectrometry: The MS/MS raw files were processed by a newly developed tag-based hybrid search engine, JUMP, which showed better sensitivity and specificity than commercial packages (e.g., Proteome Discoverer). The data was searched against the UniProt mouse database concatenated with a reversed decoy database for evaluating false discovery rate. Searches were performed using a 25-ppm mass tolerance for precursor ions and 25-ppm mass tolerance for fragment ions, fully tryptic restriction with two maximal missed cleavages, three maximal modification sites, and the assignment of a, b, and y ions. TMT tags on Lysine residues and N-termini (+229.162932 Da) were used for static modifications and Met oxidation (+15.99492 Da) was considered as a dynamic modification. MS/MS spectra were filtered by mass accuracy and matching scores to reduce protein false discovery rate to ~1%. Proteins were quantified by summing reporter ion counts across all matched PSMs using the JUMP software suite46.<br><br>Confocal microscopy: The Nikon Element software v9 was used for image acquisition and analysis.

For manuscripts utilizing custom algorithms or software that are central to the research but not yet described in published literature, software must be made available to editors/reviewers. We strongly encourage code deposition in a community repository (e.g. GitHub). See the Nature Research guidelines for submitting code & software for further information.

Data

Policy information about availability of data

All manuscripts must include a data availability statement. This statement should provide the following information, where applicable:

- Accession codes, unique identifiers, or web links for publicly available datasets
- A list of figures that have associated raw data
- A description of any restrictions on data availability

Data availability. All data supporting the findings of this study are available within the paper and the Supplementary information, including Supplementary Tables S1-S8. The RNA-seq data generated from this study has been deposited in the NCBI's Gene Expression Omnibus and is publicly accessible through GEO Series accession number GSE149637.

Field-specific reporting

Please select the one below that is the best fit for your research. If you are not sure, read the appropriate sections before making your selection.

[x] Life sciences [ ] Behavioural & social sciences [ ] Ecological, evolutionary & environmental sciences

For a reference copy of the document with all sections, see nature.com/documents/nr-reporting-summary-flat.pdf

Life sciences study design

All studies must disclose on these points even when the disclosure is negative.

Sample size	Sample size was based on trial experiments and/or previous experiments (Hunt el al. Cell Reports, 2019, PMID: 31365869)
Data exclusions	No data points were excluded.
Replication	There were no attempts at replication that failed. The figure legends report how many independent experiments were done.
Randomization	The samples were allocated to groups randomly to reduce the chance of batching effects.
Blinding	The investigators were blinded to group allocations for image analysis and quantitation, and for muscle functional measurements. For other experiments, it was not always possible to blind the experimenter to the identity of the animals.

Reporting for specific materials, systems and methods

We require information from authors about some types of materials, experimental systems and methods used in many studies. Here, indicate whether each material, system or method listed is relevant to your study. If you are not sure if a list item applies to your research, read the appropriate section before selecting a response.

Materials & experimental systems

n/a	Involved in the study	
[ ]	[x]	Antibodies
[ ]	[x]	Eukaryotic cell lines
[x]	[ ]	Palaeontology
[ ]	[x]	Animals and other organisms
[x]	[ ]	Human research participants
[x]	[ ]	Clinical data

Methods

n/a	Involved in the study	
[x]	[ ]	ChIP-seq
[x]	[ ]	Flow cytometry
[x]	[ ]	MRI-based neuroimaging

Antibodies

Antibodies used	Mouse monoclonal P4D1 anti-ubiquitin Cell SIgnaling #3936, rabbit polyclonal anti-Ref2P Abcam #178440 for Drosophila SQSTM1, rabbit monoclonal anti-tubulin (11H10) Cell Signaling #2125, rabbit monoclonal anti-GFP (D5.1) Cell Signaling #2956, rabbit polyclonal anti-UBR4 Abcam #86738, rabbit polyclonal anti-LC3 Sigma #L7543, rabbit polyclonal anti-SQSTM1/p62 Cell Signaling #5114, rabbit monoclonal anti-STUB1 (C3B6) Cell Signaling #2080, and anti-mouse and anti-rabbit HRP-linked secondary antibodies (Cell Signaling #7076 and #7074). Antibodies were diluted at 1:200 for immunostaining and at 1:1000 for western blot.
Validation	These antibodies were validated by the supplier and by us via Western blots and immunostaining on the basis of the correct MW, staining pattern, and detection or lack of detection of positive and negative genetic controls. Information on the validation of the antibodies can be found at the supplier websites and in previous publications (Hunt el al. Cell Reports, 2019, PMID: 31365869; Demontis and Perrimon, Cell, 2010, PMID: 21111239).

Eukaryotic cell lines

Policy information about cell lines

Cell line source(s)	C2C12 cells (ATCC).
Authentication	These cell lines were authenticated via phenotypic, morphological, and RNA-seq/qPCR analyses, which demonstrated a gene expression profile as expected based on previous reports.
Mycoplasma contamination	These cell lines tested negative for mycoplasma contamination and were screened regularly to avoid such possibility.
Commonly misidentified lines (See ICLAC register)	No commonly misidentified lines were used in this study.

Animals and other organisms

Policy information about studies involving animals; ARRIVE guidelines recommended for reporting animal research

Laboratory animals	Male mice with muscle-specific UBR4 knockout (mUBR4KO), controls, and wild-type C57BL6/J mice for siRNA electroporation into muscles were used at the specified age (6, 24, and 30 months of age). All mice were housed and handled in accordance with approved St. Jude Children's Research Hospital Institutional Animal Care and Use Committee protocols and fed standard chow with 12-hour light/dark cycles. Drosophila melanogaster strains are listed in Supplementary Tables S2 and S3 and were maintained at 25ºC with 60% humidity on 12-hour light/dark cycles unless otherwise specified with standard cornmeal/soy flour/yeast fly food.
Wild animals	No wild animals were used in thi study.
Field-collected samples	No field-collected samples were used in this study.
Ethics oversight	All mice were housed and handled in accordance with approved St. Jude Children's Research Hospital Institutional Animal Care and Use Committee. No approval is needed for Drosophila as this is an invertebrate organism.

Note that full information on the approval of the study protocol must also be provided in the manuscript.
