# Supplementary material for: Antagonistic control of myofiber size and muscle protein quality control by the ubiquitin ligase UBR4 during aging
Source: Nat Commun. 2021 Mar 3;12:1418. doi: 10.1038/s41467-021-21738-8 (PMC7930053; doi:10.1038/s41467-021-21738-8)
Supplement: Supplementary file 10 — Description of Additional Supplementary Files [file 41467_2021_21738_MOESM10_ESM.pdf]

Description of Supplementary Files

File name: Supplementary Data 1
Description: RNA sequencing and TMT mass spectrometry data obtained from mouse UBR4 mKO tibialis anterior muscles at 6 and 24 months of age

File name: Supplementary Data 2
Description: *Drosophila* RNAi screen data reporting the effect of RNAi for ubiquitin ligases and ubiquitin-related proteins on myofiber size, degradation of pathogenic huntingtin, and lifespan

File name: Supplementary Data 3
Description: Information on additional *Drosophila* stocks used in this study

File name: Supplementary Data 4
Description: Information on qRT-PCR oligos used in this study

File name: Supplementary Data 5
Description: TMT mass spectrometry data obtained from muscle-specific RNAi of select ubiquitin ligases in *Drosophila*

File name: Supplementary Data 6
Description: GO terms of TMT mass spectrometry data obtained from muscle-specific RNAi of select ubiquitin ligases in *Drosophila*

File name: Supplementary Data 7
Description: Comparison of protein targets modulated by UBR4 RNAi in mouse versus *Drosophila* skeletal muscle

File name: Source Data
Description: Raw data and uncropped blots for all figures
